# From Stress to Survival: Trophoblast-Derived Extracellular Vesicle Proteome Captures Aspirin-Driven Cellular Reprogramming in a Preeclampsia Model

**DOI:** 10.3390/pharmaceutics18060677

**Published:** 2026-05-29

**Authors:** Vineet Mahajan, Awanit Kumar, Jeena Jacob, Maged M. Costantine, Lauren S. Richardson, Rheanna Urrabaz-Garza, Emmanuel Amabebe, Ourlad Alzeus G. Tantengco, Ananth Kumar Kammala, Ramkumar Menon

**Affiliations:** 1 Division of Basic Science and Translational Research, Department of Obstetrics and Gynecology, The University of Texas Medical Branch at Galveston, Galveston, TX 77555, USA; vimahaja@utmb.edu (V.M.); awanitbt@gmail.com (A.K.); lestaffo@utmb.edu (L.S.R.); rhurraba@utmb.edu (R.U.-G.); emmamabe@utmb.edu (E.A.); outanten@utmb.edu (O.A.G.T.); ankammal@utmb.edu (A.K.K.); 2 Division of Maternal-Fetal Medicine, Department of Obstetrics and Gynecology, The Ohio State University College of Medicine, 395 W. 12th Ave, 5th Floor, Columbus, OH 43210, USA; maged.costantine@osumc.edu

**Keywords:** preeclampsia, low-dose aspirin, extracellular vesicles, chorion trophoblast, oxidative stress, biomarkers, angiogenesis, prophylactic window

## Abstract

**Background:** Low-dose aspirin (LDA) reduces preeclampsia (PE) risk by up to 40%, yet its molecular effects on chorion trophoblast cells (CTCs), a fetal membrane lineage at the feto-maternal interface, remain obscure. CTCs form a structural and immunoregulatory barrier whose dysfunction drives inflammation-associated membrane pathology in PE. Extracellular vesicles (EVs) released by CTCs may encode cellular stress and adaptation states, offering a molecular window into aspirin’s timing-dependent effects on PE risk modification. **Methods:** Human CTCs were challenged with cigarette smoke extract (CSE) to model oxidative stress-driven PE pathology. Two paradigms were tested: (1) prophylactic aspirin (4 and 40 µg/mL) before and/or flanking the CSE, and (2) therapeutic aspirin after the CSE challenge. The EVs were isolated via ultracentrifugation and size-exclusion chromatography, characterized by nanoparticle tracking and immunoblotting, and profiled by quantitative mass spectrometry. A network pathway analysis and machine learning biomarker selection defined the EV-encoded molecular states. **Results:** The CTC-derived EVs from the CSE-exposed cells carried a PE-like proteomic signature marked by suppressed VEGF/ECM remodeling, activated TNF-p53 apoptotic signaling, and heightened inflammation. Prophylactic low-dose aspirin shifted the EV cargo toward an EV-encoded signature consistent with preserved angiogenic potential (enrichment of VEGFA, COL1A1, and MMP14) and predicted attenuation of apoptotic and NF-κB pathway activity by an Ingenuity Pathway Analysis. High-dose aspirin produced broad transcriptional suppression without an accompanying pro-angiogenic EV signature. Therapeutic (post-injury) aspirin partially attenuated the injury-associated EV cargo but did not restore the angiogenic EV signature. An exploratory machine learning analysis of EV proteomes identified a candidate prophylactic biomarker panel anchored by HSPA8, SERPINF2, COL4A1, and PLOD1, mapped to the predicted angiogenic recovery and redox-balance pathways. These EV cargo readouts represent the predicted molecular states and require functional validation before clinical interpretation. **Conclusions:** The CTC-derived EV proteomic signatures capture the dose- and timing-dependent aspirin effects in this in vitro CTC model, positioning the chorion as a candidate pharmacological “secondary responder” favoring cellular resilience over classical anti-inflammatory suppression. As an exploratory hypothesis-generating study, EV-based molecular profiling could provide a foundation for future investigations aimed at stratifying aspirin responders from non-responders, although clinical validation in maternal plasma cohorts will be required before any translational application.

## 1. Introduction

Preeclampsia (PE), together with other placental pathologies of pregnancy, including fetal growth restriction (FGR), are major contributors to maternal and neonatal morbidity worldwide [1,2,3,4].

The placenta, specifically the trophoblast layer, is particularly vulnerable to oxidative stress, which impairs trophoblast invasion, disrupts vascular remodeling, and dysregulates immune tolerance at the feto-maternal interface [5,6].

Low-dose aspirin (LDA) prophylaxis remains the gold standard for high-risk pregnancies, reducing the incidence of preeclampsia by approximately 40% when initiated before 16 weeks of gestation [7,8,9]. However, this leaves a substantial proportion of patients as ‘non-responders,’ a clinical reality that current placenta-centric models fail to explain [10,11,12]. The exact targets of aspirin’s action, as well as its timing-dependent effects and mechanisms of action at the feto-maternal interface, remain unclear despite its widespread medical use.

The feto-maternal interface comprises two distinct compartments: the placenta/decidua and the fetal membrane/decidua, each contributing distinct functions to maintain pregnancy. Within this interface, chorion trophoblast cells (CTCs) form a key mechanical and immunological barrier, regulating biochemical exchange, ECM remodeling, and choriodecidual angiogenic signaling during pregnancy [13,14]. Dysregulation of these functions, primarily mediated by PE-associated inflammation and oxidative stress, has been implicated in adverse pregnancy outcomes, including preterm birth, which often coexists with placental dysfunction [15,16]. Interventions for adverse pregnancy outcomes have typically been developed from the mechanisms identified in placental trophoblast cells. Because CTCs share the same trophoblast lineage as placental alkaline phosphatase (PLAP)-expressing [17] placental trophoblasts and perform similar barrier and immunomodulatory functions, defining how CTCs respond to aspirin treatment is essential to improving pregnancy outcomes [18]. Understanding how CTCs respond to aspirin treatment is therefore essential to define whether aspirin confers systemic protection across all feto-maternal interfaces (placenta/decidua and choriodecidua) or acts primarily through placental pathways.

Although aspirin’s protective effects on placental endothelial and trophoblast signaling have been previously explored [19,20,21], its impact on fetal membrane trophoblasts, particularly CTCs, remains largely unexplored. Like placental villous trophoblasts, CTCs experience unique mechanical and oxidative stressors at the choriodecidual interface, where localized inflammation and extracellular vesicle (EV; small EVs: 30–200 nm, per MISEV2023 nomenclature) communication modulate tissue integrity and immune tolerance. In pregnancy, trophoblast-derived EVs are released into the maternal circulation from early gestation and play critical roles in immune modulation, angiogenesis, and spiral artery remodeling [22,23,24]. Alterations in the circulating placental EV cargo have been reported in PE [25,26] and FGR [27], establishing EVs as both potential mediators of placental pathology and candidate liquid biopsy biomarkers. While the protective effects of aspirin on placental angiogenesis are well documented, the pharmacological sensitivity of the CTC layer—the critical immunoregulatory and structural barrier at the feto-maternal interface—remains virtually uncharacterized. Importantly, CTCs express PLAP, enabling identification of CTC-derived EVs in maternal circulation through PLAP-based capture strategies [28]. The choriodecidual interface exposes CTCs to specific mechanical forces and oxidative stress because they exist in a distinct environment where local inflammation and EV communication systems affect tissue stability and immune acceptance. The timing of aspirin exposure in relation to inflammatory or oxidative stress remains a critical question for maternal–fetal pharmacology. The protective effects of aspirin are thought to emerge from its administration, which creates an anti-inflammatory state through COX-dependent eicosanoid signaling and NF-κB suppression, following pre-inflammatory stressor administration [29,30]. The protective effect of preconditioning helps maintain chorionic cell survival and supports the production of angiogenic factors and provides membrane stability. In contrast, therapeutic aspirin treatment, where aspirin is introduced after an insult, may act through secondary mechanisms limiting damage propagation, restoring redox balance, or promoting reparative signaling [31,32].

Differentiating these timing-dependent effects within CTCs is critical, because aspirin is often initiated at variable gestational stages in clinical practice, and its prophylactic versus therapeutic efficacy may diverge between the placental and fetal membrane compartments. Given the central role of the chorion in maintaining fetal–maternal barrier integrity, deciphering aspirin’s direct and EV-mediated effects on CTC signaling, angiogenesis, apoptosis, and inflammation is essential. Addressing this question will (1) define whether aspirin’s benefits extend beyond the placenta to the fetal membrane; (2) clarify whether prophylactic and therapeutic exposures elicit distinct molecular outcomes; and (3) inform and identify gestational timing strategies to optimize aspirin use for reducing both preeclampsia and preterm birth.

This study investigates how low-dose aspirin, when administered either before (prophylactic/risk exposure) or after (therapeutic) an oxidative injury, alters the proteomic and functional landscape of CTC-derived EVs. We postulate that the chorion functions not merely as a passive bystander, but as a distinct ‘secondary responder’ tissue. Here, we investigate whether the EV cargo derived from CTCs captures distinct molecular states corresponding to oxidative injury, partial recovery, and stress-adaptive resilience. Because EVs integrate molecular signals across time and stress states, they offer a unique window into cellular adaptation that cannot be captured by single time-point cytokine or transcript measurements. By profiling the EV cargo, we can resolve whether trophoblasts encode injury, partial recovery, or stress-tolerant adaptive programs in response to oxidative challenge. Thus, EVs serve not merely as biomarkers but as molecular records of stress history and adaptive capacity at the feto-maternal interface. Aspirin exposure is employed here not as a therapeutic endpoint but rather as a pharmacologic perturbation to reveal how EV proteomic composition reflects the timing of stress exposure and adaptive signaling. By integrating EV proteomics with network-based and machine learning analyses, we define the EV-encoded molecular states that distinguish injury-dominant from stress-resilient trophoblast responses, providing a framework for EV-based biomarker discovery at the feto-maternal interface.

## 2. Materials and Methods

### 2.1. Cell Source and Culture Conditions

CTCs were isolated in-house from fetal membrane tissues obtained from John Sealy Hospital at University of Texas Medical Branch (UTMB), Galveston, TX, USA. Tissues were collected under IRB-approved exempt protocols for discarded and de-identified specimens from uncomplicated term Cesarean deliveries. Isolated CTCs were immortalized and maintained as previously described. All experiments described in this study used single immortalized CTC line derived from one uncomplicated term donor; biological replicates (n = 3) refer to independent passages and independent treatment runs of this line rather than independent donor isolations. This is acknowledged as limitation in Section 4.9. Cells were cultured in 1:1 mixture of DMEM and Ham’s F-12 medium (Corning, Manassas, VA, USA; Ref# 10-092-CV) supplemented with 0.2% fetal bovine serum, 0.1 mM 2-mercaptoethanol, 0.5% penicillin–streptomycin, 0.3% BSA, 1% ITS-X supplement, 2 μM CHIR99021, 0.5 μM A83-01, 1 μM SB431542, 1.5 μg/mL L-ascorbic acid, 50 ng/mL EGF, 0.8 mM valproic acid, and 5 μM Y-27632. Cells were subcultured at low density (approximately 2 × 10^6^ cells per T175 flask) to avoid confluence at time of harvest.

### 2.2. Immunocytochemistry

CTC identity was confirmed by immunocytochemistry for cytokeratin-7 (CK7) and placental alkaline phosphatase (PLAP). After 24 h of subculture, cells were fixed with 4% paraformaldehyde, permeabilized with 0.5% Triton X-100, and blocked with 3% BSA in PBS. Cells were incubated overnight at 4 °C with anti-CK7 (ab9021, Abcam, Cambridge, UK) and anti-PLAP (ab243731) antibodies (1:200). Following PBS washes, secondary antibodies anti-mouse Alexa Fluor 488 (ab150073) and anti-rabbit Alexa Fluor 594 (ab150080) were applied (1:1000, 1 h, room temperature, dark). Nuclei were counterstained with DAPI and mounted using MoWiol 4-88 (Sigma-Aldrich, St. Louis, MO, USA; Cat.# 81381). Images were acquired on Keyence fluorescence microscope (Itasca, IL, USA).

### 2.3. Cigarette Smoke Extract (CSE) Preparation

Water-soluble CSE was prepared as previously reported and diluted 1:50 in culture medium immediately prior to use to induce oxidative stress [33,34].

### 2.4. Therapeutic Aspirin Treatment

Based on the reported plasma Cmax of low-dose aspirin in pregnancy, 4 μg/mL and 40 μg/mL doses were selected [35]. The 4 μg/mL concentration approximates the reported total (free + protein-bound) plasma salicylate Cmax achieved after a single oral 81–100 mg low-dose of aspirin in pregnant women after the rapid hepatic deacetylation of aspirin to its active salicylate metabolite. Because aspirin acetylates COX irreversibly within minutes of absorption, in vitro exposure to 4 μg/mL acetylsalicylic acid was used to model the brief pharmacologically active window experienced at the feto-maternal interface following systemic dosing. The 40 μg/mL concentration represents a 10-fold supratherapeutic exposure that is not directly clinically achievable with low-dose aspirin but approximates the plasma salicylate ranges seen with higher-dose anti-inflammatory regimens (~325–650 mg), and is included as a mechanistic ceiling to expose the dose saturation effects on COX-1/COX-2 selectivity. Tissue-level exposure at the choriodecidual interface is not directly measurable; therefore, both concentrations should be interpreted as in vitro pharmacological probes spanning the therapeutic-to-supratherapeutic range rather than direct equivalents of any prescribed clinical dose. Throughout the manuscript, “low dose” and “high dose” refer to the in vitro working concentrations only. The aspirin (Sigma-Aldrich, St. Louis, MO, USA, Cat#1044006) stock was prepared at 10 mg/mL in DMSO and diluted in medium to the working concentrations. The final DMSO concentration was held constant at 0.4% (*v*/*v*) across all treatment groups, including the controls and aspirin-free conditions, to exclude vehicle-related effects. The CTCs were first exposed to CSE for 24 h (days 1–2), followed by addition of aspirin (days 2–3) with or without continued CSE. The control conditions included media alone, equivalent DMSO, and aspirin alone. After 24 h of aspirin treatment, the conditioned culture media (CCM) and cell lysates were collected. The cells were lysed in 1× RIPA buffer containing protease and phosphatase inhibitors and stored at −20 °C.

### 2.5. Prophylactic Aspirin Treatment

For the prophylactic studies, the CTCs were treated with aspirin (4 or 40 μg/mL) for 24 h after subculture. The cells were then challenged with CSE (1:50) in the presence or absence of the same aspirin dose for an additional 48 h. A continuous exposure arm (Asp → CSE → Asp) was included. The controls received equivalent DMSO. The CCM and lysates were harvested as above.

### 2.6. Cytokine Measurement and Inflammation Index

The IL-6, IL-8, IL-10, and TNF concentrations in the CCM were quantified using multiplex immunoassays according to the manufacturer’s instructions MILLIPLEX^®^ Human Cytokine/Chemokine/Growth Factor Panel A (MilliporeSigma, Burlington, MA, USA; Cat. No. HCYTA-60K)). To evaluate the net inflammatory balance, a composite Inflammation Index was calculated for each replicate as z(IL-6) + z(IL-8) + z(TNF) − z(IL-10). The IL-6/IL-10 and TNF/IL-10 ratios were used as secondary metrics. For the groupwise analyses and figure generation, individual biological replicates were assigned to the treatment categories based on the experimental sequence rather than numeric dose labels. All the samples annotated as CSE+Asp1, CSE+Asp2, and CSE+Asp3—representing CSE exposure followed by aspirin treatment at either 4 or 40 μg/mLwere pooled into a single Asp+CSE group, as these conditions reflect the same biological paradigm of aspirin administered after oxidative stress. The samples receiving aspirin before and after the CSE challenge (Asp → CSE → Asp) were designated as the “Continuous” group. The controls included Ctrl (untreated) and CSE alone; the wells treated with aspirin in the absence of CSE were classified as Asp alone. The composite Inflammation Index and IL-6/IL-10 ratios were calculated for each replicate and summarized as the mean ± SEM within these predefined groups (n = 3 per group). This grouping scheme was applied uniformly to both the therapeutic and prophylactic datasets to enable direct comparison of aspirin timing effects.

### 2.7. Cytotoxicity and Viability

Aspirin cytotoxicity (0–100 μg/mL) was assessed using LDH release (Abcam ab197004). Ten microliters of media were mixed with 90 μL LDH reaction mix, incubated for 10 min, and fluorescence was measured at Ex/Em 535/587 nm. Viability was assessed using Alamar Blue (Invitrogen, Thermo Fisher Scientific, Waltham, MA, USA); resazurin conversion was measured at Ex/Em 560/590 nm after 3 h incubation.

### 2.8. NF-κB and p38 MAPK Signaling

Protein levels of total and phospho-NF-κB and p38 MAPK were measured using Simple Western JESS™ (Protein Simple, San Jose, CA, USA). Lysates (0.2 mg/mL) were analyzed with antibodies: NF-κB (#4764S), phospho-NF-κB (S536), p38 MAPK (#9212), and phospho-p38 MAPK (#4511) (Cell Signaling Technology, Danvers, MA, USA; 1:50). Detection used anti-rabbit chemiluminescent module (Bio-Techne DM001) and Compass software v6.3.

### 2.9. Extracellular Vesicle Isolation and Characterization

EVs were isolated from approximately 25–30 mL of CCM by differential centrifugation, 100 kDa ultrafiltration, 0.2 μm filtration, and ultracentrifugation at 100,000× *g* for 2 h at 4 °C, followed (pooled from 5 to 6 T175 flasks per condition) by ExoSpin purification. CTC culture medium was prepared with EV-depleted FBS (overnight ultracentrifugation at 100,000× *g*, 4 °C) to minimize bovine EV contamination. Final EV pellets were resuspended in 100–150 μL sterile PBS. Total EV protein yield, measured by micro-BCA, ranged from approximately 8–15 μg per condition (n = 3 biological replicates per group). Particle-to-protein ratio averaged 2–5 × 10^9^ particles/μg across preparations, consistent with values typically reported for moderately to highly pure small EV preparations under MISEV2023 reporting guidelines. Representative size distribution histograms from ZetaView are provided in Supplementary Figure S4. Particle size and concentration were determined by ZetaView PMX 110 (Particle Metrix). EV protein markers (PLAP, FLOT-1, and CD9) were assessed using Simple Western JESS as above. Per MISEV2023, at least one transmembrane protein (CD9) and one cytosolic-recovered protein (FLOT-1) were assessed [36]. PLAP was included as tissue-of-origin marker to confirm trophoblast derivation of isolated EVs. We have submitted all relevant data from our experiments to EV-TRACK knowledgebase36 (EV-TRACK ID: EV260015), which are currently being processed.

### 2.10. NanoLC-MS/MS Proteomics

EV peptides were analyzed on UltiMate 3000 RSLCnano (Thermo Fisher Scientific, Waltham, MA, USA) coupled to Orbitrap Eclipse (Thermo Fisher Scientific, Waltham, MA, USA) using DIA with staggered 8-Da windows (400–900 *m*/*z*). Data were processed in FragPipe v22.0 with MSFragger/DIA-NN against UniProt Homo sapiens (5 February 2025). FDR was set to 2%; differential expression used limma with Benjamini–Hochberg correction (adjusted *p* < 0.05; |log_2_FC| ≥ 1). Proteomics was performed on EV preparations from each biological replicate analyzed individually (no pooling); thus, each treatment group is represented by three independent EV proteomes that were processed and quantified in parallel.

### 2.11. Proteomics Pathway Analysis Using Ingenuity Pathway Analysis (IPA)

**Figure 10:**
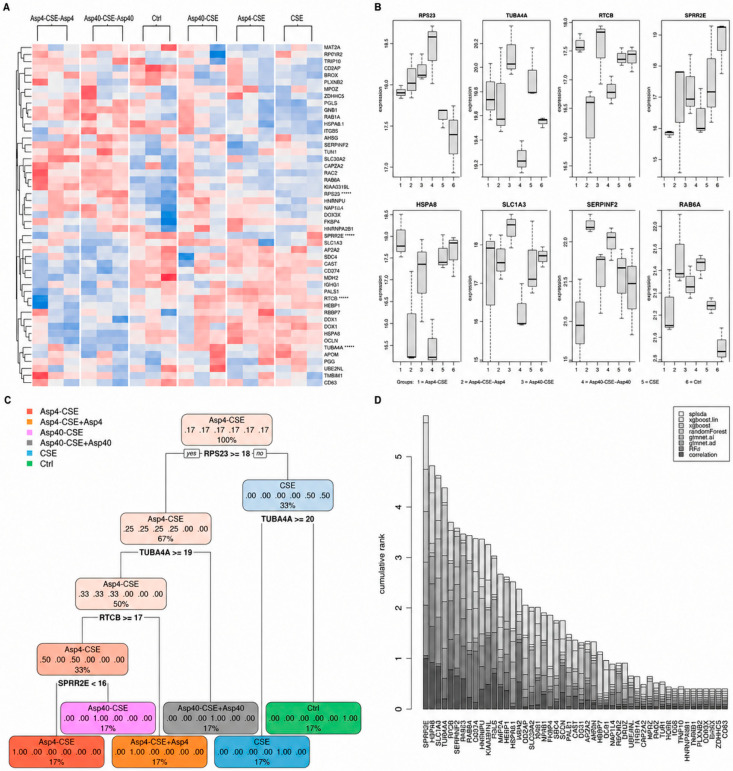
Machine learning integration of EV proteomics reveals a prophylactic aspirin-associated biomarker signature. (**A**) Heatmap of ML-predicted biomarker proteins identified by ensemble feature selection (LASSO, random forest, elastic net, and PLS-DA) across prophylactic treatment conditions: CSE+Asp4, Asp40-CSE+Asp40, Ctrl, Asp40-CSE, Asp4-CSE, and CSE. Unsupervised hierarchical clustering of columns shows CSE+Asp4 and Asp40-CSE+Asp40 clustering together, Asp40-CSE and Asp4-CSE clustering together, and Ctrl and CSE as distinct groups. Color scale: blue (low, −3.15) to red (high, +2.91), representing z-scored protein abundance. Proteins marked with ***** indicate features showing strong statistical significance across groups (**B**) Box plots validating ML-predicted protein expression across treatment groups for eight representative biomarkers: RPS23, TUBA4A, RTCB, SPRR2E (upper row), HSPA8, HNRNPU, SLC1A3, and SERPINF2 (lower row). Boxes represent interquartile range; horizontal lines indicate median; whiskers denote data range. Expression patterns demonstrate strong alignment between ML-predicted and experimentally validated protein abundance across prophylactic conditions. (**C**) Decision tree classification map showing the biomarker thresholds that separate prophylactic CTC treatment groups. Node colors indicate the predicted class, and terminal nodes show the final classification probability and proportion of samples assigned to each group. (**D**) Cumulative biomarker rank plot showing the relative contribution of selected genes across multiple machine learning feature selection approaches. Higher cumulative rank indicates stronger and more consistent biomarker importance across the evaluated ML algorithms. Asterisks indicate statistical significance.

**Figure 11:**
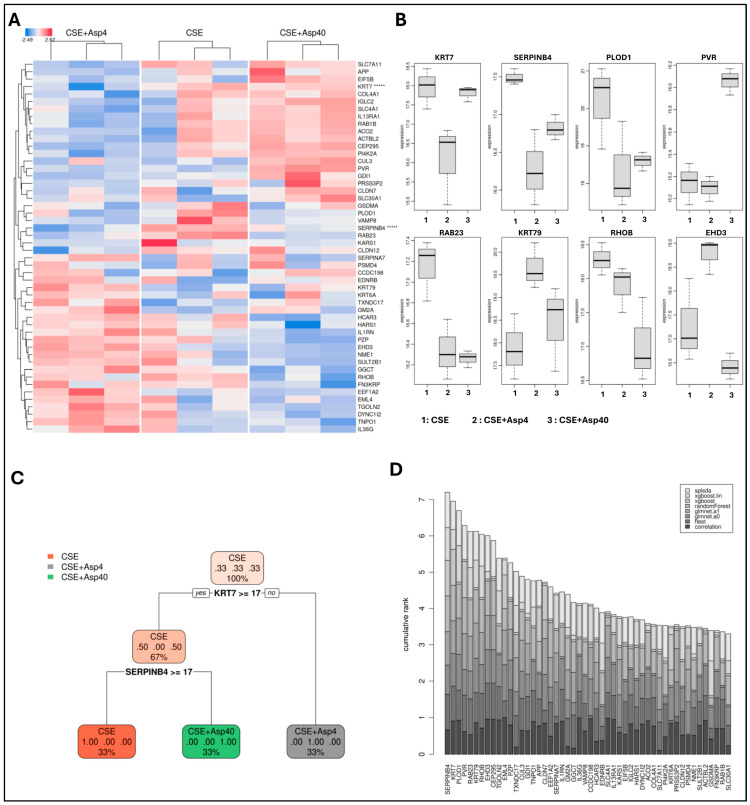
Machine learning identification and proteomic validation of aspirin-modulated biomarker signatures after CSE exposure. (**A**) Heatmap of predicted biomarker proteins identified using a machine learning-based feature selection approach, highlighting dose-dependent molecular signatures across CSE+Asp4, CSE, and CSE+Asp40 conditions. Values are scaled to show relative abundance (blue, low; red, high), and unsupervised hierarchical clustering reveals distinct aspirin-dependent biomarker patterns. Proteins marked with ***** indicate BigOmics-reported statistically significant features among the ML-prioritized biomarker panel (**B**) Proteomic validation of selected ML-predicted biomarkers shown as box plots, confirming differential expression of representative proteins (e.g., KRT7, SERPINB4, PLOD1, PVR, RAB23, KRT79, RHOB, and EHD3) across treatment groups. Boxes indicate median and interquartile range; whiskers denote data range. Supplementary Figure S1: IPA identifies distinct signaling networks driving prophylactic aspirin-induced cell survival. (**C**) Decision tree classification map showing the biomarker thresholds that separate therapeutic CTC treatment groups. Node colors indicate the predicted class, and terminal nodes show the final classification probability and proportion of samples assigned to each group. (**D**) Cumulative biomarker rank plot showing the relative contribution of selected therapeutic CTC biomarkers across multiple machine learning feature selection approaches. A higher cumulative rank indicates stronger and more consistent biomarker importance across the evaluated algorithms. Asterisks indicate statistical significance.

Differentially expressed proteins identified from DIA proteomics were further interrogated using Ingenuity Pathway Analysis (IPA, winter 2025, spring 2026 release, Qiagen, Redwood City, CA, USA) to determine enriched biological functions, canonical pathways, and upstream regulators [37]. Protein identifiers with corresponding log_2_ fold changes and adjusted *p*-values were uploaded into IPA, and the Core Analysis module was performed using the Ingenuity Knowledge Base (human reference). Activation or inhibition of pathways and regulators was inferred using z-score algorithm, with significance determined by right-tailed Fisher’s exact test (*p* < 0.05). Networks related to inflammatory signaling, oxidative stress, autophagy, and extracellular vesicle biogenesis were prioritized based on relevance to CTC biology and aspirin response. Given small sample size (n = 3 biological replicates per group), ML analysis was explicitly designed and is reported as exploratory, hypothesis-generating biomarker discovery rather than predictive model development. To reduce overfitting risk and limit dimensionality before biomarker ranking, features were first filtered based on differential expression criteria, including log_2_ fold change and/or statistical significance. Candidate biomarkers were then prioritized using biomarker analysis module, which applies multiple machine learning and statistical feature-ranking approaches, including sparse partial least squares, glmnet regularized regression, random forest, extreme gradient boosting, F-test, and correlation-based ranking. Biomarkers were prioritized based on cumulative ranking across methods rather than dependence on single algorithm. Class balance was preserved by analyzing groups with equal numbers of biological replicates in each pairwise comparison. Because no independent validation cohort was available and number of samples per group was very small, model performance metrics and decision tree classifications were interpreted only as exploratory indicators of group separation and not as estimates of clinical predictive performance. Candidate biomarkers were further evaluated in context of IPA pathway enrichment, predicted upstream regulators, mechanistic networks, cytokine profiles, and Inflammation Index data to support hypothesis-driven biological interpretation. Given very small n, these should not be interpreted as estimates of clinical predictive performance, which will require independent validation in larger, longitudinal patient cohorts. Additional details of decision map and model score are included in Figure 10 and Figure 11. Predicted upstream regulators and mechanistic networks were integrated with cytokine and Inflammation Index data to generate hypothesis-driven models.

### 2.12. Omics Playground for Integrative Visualization and Biomarker Discovery

Raw protein abundance matrices generated from FragPipe/DIA-NN were imported into Omics playground (BigOmics, Lugano, Switzerland) for complementary exploratory analysis [38,39]. Data were log_2_-transformed, normalized and median-centered prior to analysis. The platform was used to perform principal component analysis (PCA), hierarchical clustering, biomarker discovery and pathway enrichment based on Reactome and Gene Ontology databases. ML-based biomarker discovery was performed using Omics Playground (BigOmics Analytics, Switzerland) [21]. Ensemble analysis of LASSO, elastic net, random forest, and PLS-DA was applied to identify proteins most strongly discriminating against CSE vs. Asp+CSE and continuous groups. Model performance was assessed by k-fold cross-validation with AUC-ROC. Top candidate biomarkers were cross-validated with IPA-derived pathways to prioritize EV proteins potentially mediating aspirin-dependent modulation of CTC responses. ML candidates were filtered for protein secretion/shedding potential, known plasma detectability, commercial immunoassay availability, FBS contamination risk, and trophoblast/placental expression evidence.

### 2.13. Statistical Analysis

Experiments included three biological replicates (n = 3). Groupwise Inflammation Index and cytokine ratios were summarized as mean ± SEM. One-way ANOVA with appropriate post hoc tests was performed using GraphPad Prism version 11; *p* < 0.05 was considered significant.

## 3. Results

**Figure 1:**
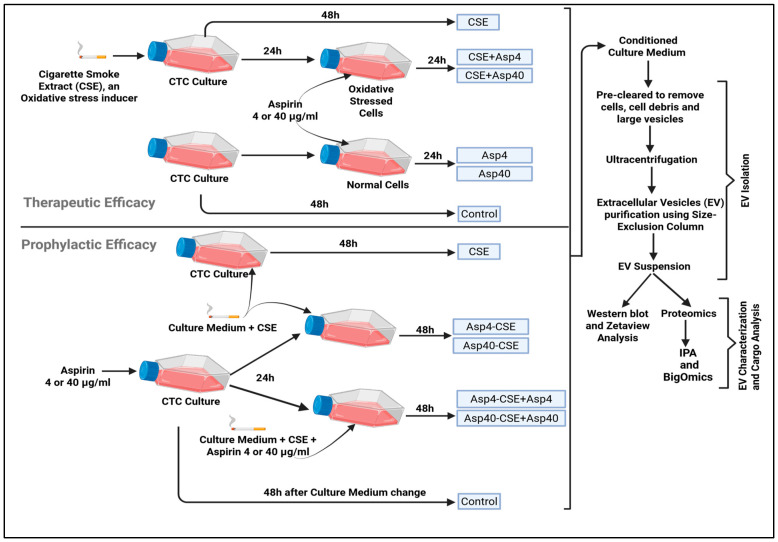
Experimental design illustrating therapeutic and prophylactic aspirin efficacy in oxidative stress-induced chorion trophoblast cells and downstream EV analysis. CTCs are cultured and exposed to cigarette smoke extract (CSE) to induce oxidative stress. Upper panel (therapeutic efficacy): CTCs are first subjected to CSE for 24 h, followed by treatment with aspirin (4 or 40 µg/mL; Asp4 or Asp40) for an additional 24 h. Parallel groups include CSE only, aspirin only (Asp4 or Asp40), and untreated control conditions. Lower panel (prophylactic efficacy): CTCs are co-treated with aspirin and CSE for 48 h (Asp4-CSE, Asp40-CSE), or pre-treated with aspirin for 24 h prior to CSE exposure with continued aspirin (Asp4-CSE-Asp4, Asp40-CSE-Asp40) for 48 h. Conditioned culture media are collected 48 h after treatment or medium change, depending on group allocation. Right panel (EV workflow): Conditioned media are sequentially pre-cleared to remove cells, debris, and large vesicles, followed by ultracentrifugation and EV purification using size-exclusion chromatography. Purified EVs are characterized by ZetaView nanoparticle tracking analysis and Western blotting, and EV cargo is analyzed using quantitative proteomics followed by Ingenuity Pathway Analysis (IPA) and Omics Playground machine learning. This experimental framework enables comparative assessment of aspirin’s therapeutic versus prophylactic effects on oxidative stress responses and EV-mediated signaling at feto-maternal interface.

The oxidative stress-associated cellular changes and sterile inflammatory response mimicking the reported pathologies of PE were recreated using CTCs after treating them with cigarette smoke extract (CSE), a well-established oxidative stress inducer. This study incorporated two distinct experimental paradigms: a prophylactic arm, in which aspirin was administered before and after the oxidative stress exposure, and a therapeutic arm, in which aspirin was administered only after the onset of oxidative stress-induced injury in the CTCs. This study tested two prophylactic strategies and one treatment option in vitro. The prophylactic strategies were as follows: (1) Asp (4 [low dose] and 40 µg/mL [high dose]) followed by CSE treatment (Asp4–CSE-Asp4, Asp40-CSE-Asp40); (2) Asp followed by CSE treatment and continuing treatment with Asp [CSE-Asp 4 (4 µg/mL) and CSE-Asp40 (40 µg/mL)], as outlined in Figure 1. A treatment condition was also tested, where exposure to CSE was followed by treatment (CSE-ASP; both at 4 and 40 µg/mL). The data detailed below are based on our observations of the EVs released from the CTCs after exposure or treatment with the intent of developing trophoblast EVs (PLAP+ve) as a biomarker of feto-placental well-being during pregnancy. For clarity and ease of discussion, the abbreviations in parentheses will be used in the subsequent sections.

### 3.1. Cell Characteristics

CTCs express trophoblast markers and produce PLAP^+^ EVs across baseline and all treatment conditions.

**Figure 2:**
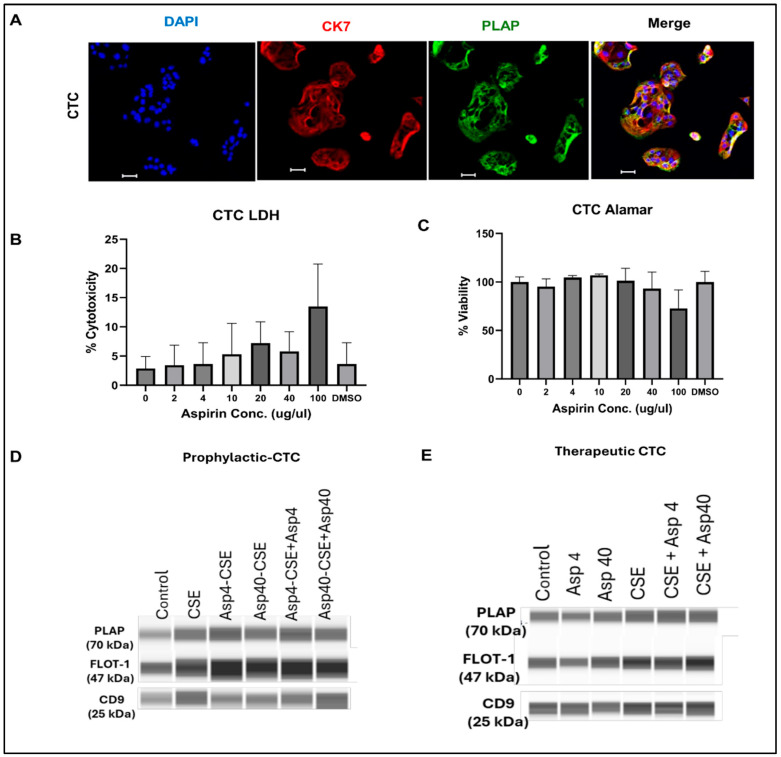
Cell characterization and aspirin sensitivity. (**A**) Immunocytochemistry confirming co-expression of CK7 (red) and PLAP (green) in chorion trophoblast cells (CTCs), with DAPI nuclear counterstain (blue). Merged images confirm trophoblast identity. Scale bar: 50 µm. (**B**) LDH cytotoxicity assay across aspirin concentrations (0–100 µg/mL). Cytotoxicity remains < 5% at 2–40 µg/mL, with modest increase (~15%) at 100 µg/mL. DMSO vehicle control shows no cytotoxicity. (**C**) Alamar Blue viability assay: Viability is maintained at ~100% across 2–40 µg/mL, with slight reduction at 100 µg/mL. (**D**) Western blotting of EVs isolated from prophylactic treatment conditions (control, CSE, Asp4-CSE, Asp40-CSE, Asp4-CSE-Asp4, and Asp40-CSE-Asp40), confirming expression of PLAP (70 kDa), FLOT-1 (47 kDa), and CD9 (25 kDa). (**E**) Western blotting of EVs from therapeutic conditions (control, Asp4, Asp40, CSE, CSE+Asp4, and CSE+Asp40), confirming PLAP, FLOT-1, and CD9 expression. PLAP detection in EVs from all conditions confirms trophoblast origin. Data represent mean ± SEM of n = 3 biological replicates.

To validate the trophoblast phenotype of the CTCs, we first performed immunocytochemical and functional characterization assays prior to any CSE or aspirin treatment. The CTCs showed distinct expression of trophoblast markers, with strong cytoplasmic staining for CK7 and membrane-associated expression of placental alkaline phosphatase (PLAP) (Figure 2A). The cells formed cohesive epithelial monolayers with a uniform nuclear morphology, confirming a stable trophoblast identity.

Next, we assessed the baseline cell viability and cytotoxicity across a range of aspirin concentrations to confirm dose tolerability. The LDH assays showed low cytotoxicity across all doses tested, with values remaining near the baseline up to 40 μg/mL, and only mild elevation at 100 μg/mL (Figure 2B). The Alamar Blue assays showed cell viability (>90%) across all aspirin concentrations, indicating that CTCs tolerate low-to-mid-range dosing without loss of metabolic activity (Figure 2C).

Western blotting of EVs from all prophylactic conditions (Figure 2D,E) and all therapeutic conditions (Figure 2E) confirmed expression of EV markers FLOT-1 (47 kDa) and CD9 (25 kDa). Critically, PLAP (70 kDa) was detected in the EVs from every treatment condition in both experimental arms, confirming that CTC-derived EVs retain their trophoblast origin identity regardless of CSE or aspirin exposure.

### 3.2. EV Characterization Under Control and CSE Exposure

The EVs were isolated from the conditioned media of all treatment groups by sequential ultracentrifugation and SEC purification, and characterized in accordance with MISEV2023 guidelines. The EVs released by the CTCs under baseline and CSE-exposed conditions were characterized using a nanoparticle tracking analysis to define vesicle abundance and size distribution prior to the downstream proteomic assessment. Across all conditions, the EV preparations displayed size profiles consistent with small EVs (sEVs) as defined by MISEV2023; the term “exosome” is avoided throughout because the biogenesis pathway was not directly verified, with modal diameters ranging from 100 to 150 nm.

In the prophylactic dataset (summarized in Table 1), the EVs from untreated CTCs exhibited a mean concentration of 2.30 × 10^10^ particles/mL and a mean diameter of 119.5 nm, representing the typical vesicle population produced by unstressed trophoblasts. The EVs collected after CSE exposure showed a concentration of 4.50 × 10^10^ particles/mL with a mean diameter of 105.2 nm, reflecting a shift toward a higher number of small EVs within the secreted population.

In the therapeutic dataset (summarized in Table 2), the EV concentration remained within the same order of magnitude across conditions (ranging from 2.10 × 10^10^ to 3.50 × 10^10^ particles/mL), and the mean EV diameters (approximately 135–142 nm) met the MISEV2023 definition of small EVs (sEVs). These measurements indicate that CTCs maintain stable EV biophysical profiles during sustained exposure, with particle abundance varying within physiological ranges.

### 3.3. CSE Exposure Establishes a Preeclampsia-like Baseline Injury State and Rewires the CTC-EV Proteome Toward Apoptosis, Coagulation, and Vascular Suppression

**Figure 3:**
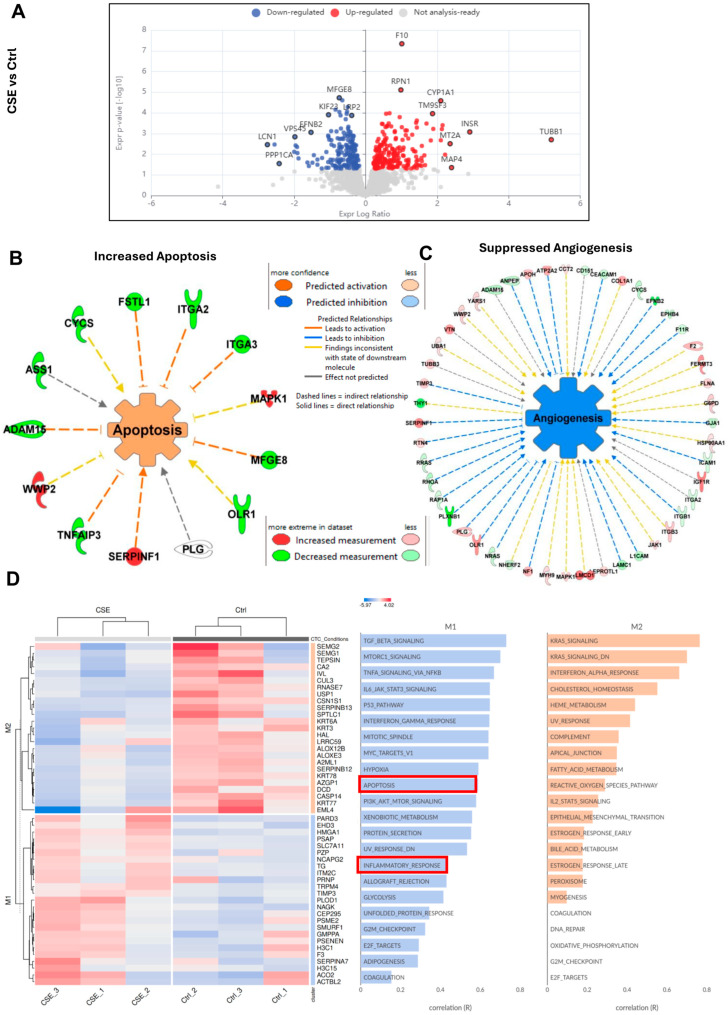
Oxidative stress rewires CTC-EV proteomic signatures toward cell death and vascular suppression. (**A**) Volcano plot showing differential protein expression in CSE versus control CTC-EVs. Red: upregulated; blue: downregulated; grey: not significant or not analysis ready. F10 (Coagulation Factor X) is the most significantly upregulated protein; MFG-E8 (Lactadherin) is among the most significantly downregulated pro-angiogenic proteins. (**B**) IPA functional network predicting increased apoptosis in CSE-derived EVs. (**C**) IPA functional network predicting reduced angiogenesis in CSE-derived EVs. (**D**) Left: Hierarchical clustering of differentially expressed EV proteins between CSE and control conditions, showing clear sample segregation. Right: Pathway enrichment analysis. Module 1 (M1, blue bars): Pathways positively correlated with the CSE-altered proteome, including TGF-β, mTORC1, TNFα/NF-κB, IL-6/JAK/STAT3, p53, apoptosis (red box), PI3K/AKT/mTOR, and inflammatory response (red box). Module 2 (M2, orange bars): Associated pathways, including KRAS, complement, cholesterol homeostasis, reactive oxygen species, and coagulation.

To establish a baseline injury state for the subsequent treatment comparisons, we first examined differential expression of proteins between the untreated control and CSE-exposed CTC-derived EVs (Figure 3A). Exposure to CSE drove a distinct shift in the EV cargo composition. Among the most prominently upregulated proteins, Coagulation Factor X (F10) stood out as the single most significantly enriched hit, pointing toward a pro-coagulant EV phenotype under oxidative stress. This was accompanied by upregulation of Ribophorin I (RPN1), an endoplasmic reticulum stress marker, and CYP1A1, a well-characterized xenobiotic metabolism enzyme and classical CSE-responsive gene. Additional upregulated cargo included the cytoskeletal stress marker Tubulin β1 (TUBB1), the metabolic signaling receptor INSR, and the microtubule-associated protein MAP4, collectively suggesting broad cellular stress responses imprinted onto the EV proteome.

Conversely, several proteins with key homeostatic and pro-angiogenic roles were significantly depleted in the CSE-derived EVs. Most notably, Milk Fat Globule EGF Factor 8 (MFG-E8/Lactadherin), a glycoprotein critically involved in efferocytosis, angiogenesis, and vascular homeostasis, was markedly downregulated. Similarly, Ephrin-B2 (EFNB2), a regulator of vascular development, and VPS4B, an ESCRT pathway component central to EV biogenesis itself, were significantly reduced. The losses in Kinesin Family Member 2B (KIF2B), Lipocalin-1 (LCN1), and protein phosphatase PPP1CA further reflect disruption of intracellular transport, lipid metabolism, and signaling, painting a picture of trophoblasts under duress, shedding the EVs that carry signatures of injury while losing their protective and regenerative capacity. Taken together, these changes suggest that oxidative stress fundamentally reshapes the trophoblast EV proteome, enriching the damage-associated cargo while stripping away the protective and pro-angiogenic factors.

Notably, the PLAP-expressing CTCs exhibited molecular alterations comparable to those previously reported in placental trophoblast cells, indicating that the fetal membrane compartment itself manifests PE-like injury signatures. This finding suggests that fetal membrane-derived trophoblasts and their EVs may contribute directly to PE pathophysiology, and may represent an additional target for therapeutic intervention.

The IPA functional analysis predicted increased apoptosis based on the network of CSE-altered proteins (Figure 3B). The apoptosis network incorporated multiple convergent pro-death signals, consistent with CSE-induced trophoblast injury. Simultaneously, the IPA predicted reduced angiogenesis following CSE exposure (Figure 3C). The anti-angiogenic network included downregulation of pro-vascular proteins and loss of growth factor signaling, indicating that CSE-damaged CTC-EVs carry cargo that suppresses vascular function at the feto-maternal interface. Consistent with oxidative injury, CSE exposure strongly activated apoptotic programs, reflected by increased abundance of CASP3, BAX, and multiple p53-regulated stress response proteins. The proteomic and network-level analyses of CTC-derived EVs revealed that CSE exposure activated TNF-driven apoptotic signaling and p53-dependent cellular stress pathways (Supplementary Figure S1A,B), aligning with established mechanisms of PE-related trophoblast injury. In parallel, CSE markedly suppressed angiogenic and ECM-remodeling proteins, including VEGFA, MMP14, and COL4A1, indicating loss of endothelial support and diminished tissue-remodeling capacity. This molecular pattern closely parallels the angiogenic insufficiency and impaired vascular remodeling characteristic of PE-affected placental and fetal membrane tissues.

To move beyond the individual protein changes and understand the broader biological programs at play, we performed a pathway enrichment analysis of differentially expressed proteins between the CSE-treated and control CTC-derived sEVs. The hierarchical clustering revealed two major coordinated expression modules (Figure 3D).

The first module (M1), representing the pathways activated under CSE exposure, was dominated by inflammatory and cell death signaling. TNFα signaling via NF-κB and IL-6/JAK/STAT3 signaling featured prominently, pointing to a distinct pro-inflammatory program embedded within the EV cargo. Alongside these, activation of the p53 pathway and apoptotic signaling indicated that the CSE-challenged trophoblasts were not merely inflamed but actively primed for cell death, a finding consistent with the tissue injury phenotype observed in preeclampsia. The interferon-γ response, PI3K/AKT/mTOR signaling, and hypoxia response pathways were also enriched, reflecting the convergence of immune activation, survival signaling, and oxygen-sensing mechanisms that typify a stressed placental microenvironment. Notably, enrichment of allograft rejection and TGF-β signaling pathways hinted at disrupted immune tolerance at the feto-maternal interface, a hallmark of preeclampsia pathogenesis.

The second module (M2) captured a set of contextual metabolic and homeostatic pathways that, while not classically inflammatory, are increasingly recognized as contributors to placental dysfunction. The KRAS signaling and interferon-α response suggested altered proliferative and innate immune signaling, while enrichment of cholesterol homeostasis, fatty acid metabolism, and heme metabolism pointed to broader metabolic rewiring under oxidative stress. Particularly striking was the enrichment of the reactive oxygen species pathway and complement cascade, both of which directly reinforce the oxidative and immunological damage initiated by the CSE. The coagulation pathway enrichment in M2 aligned with the striking upregulation of Factor X (F10) observed at the individual protein level, reinforcing a coherent pro-thrombotic EV signature.

Taken together, these two modules paint a picture of trophoblast-derived EVs that carry not just isolated stress markers but an integrated, multi-layered program of inflammation, apoptosis, metabolic disruption, and coagulation, effectively serving as molecular messengers of fetal membrane distress and oxidative stress (Table 3).

### 3.4. Prophylactic Aspirin Co-Treatment Reduces Apoptosis in a Dose-Dependent Manner and Restores Angiogenesis Exclusively at a Low Dose

**Low-dose co-treatment (Asp4-CSE vs. CSE):** As angiogenesis impairment is a major pathology associated with PE, we first examined how prophylactic aspirin influences angiogenic signaling within CTCs, using the EV cargo as a proxy for the underlying cellular processes.

**Figure 4:**
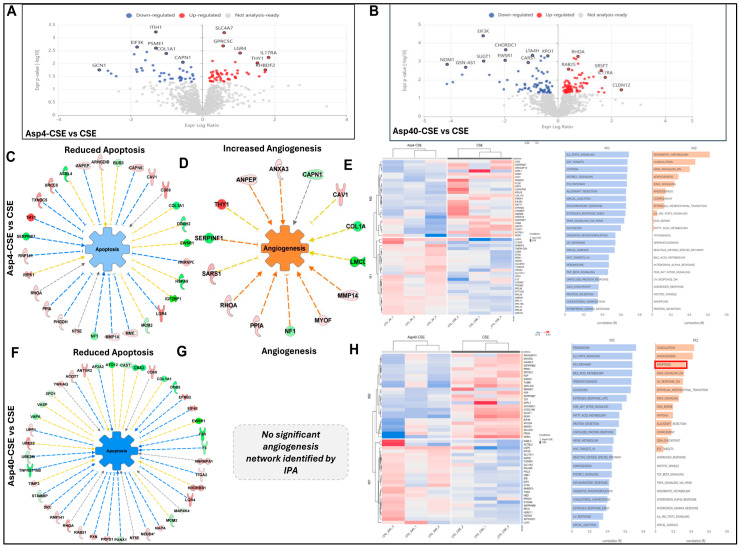
Prophylactic aspirin co-treatment reprograms CSE-induced trophoblast EV protein expression in a dose-dependent manner. (**A**) Volcano plot: Asp4-CSE vs. CSE. Significantly upregulated proteins include SLC4A7, LGR4, GPRC5C, IL17RA, THY1, and RHBIDF2. (**B**) Volcano plot: Asp40-CSE vs. CSE. Fewer significant changes, including CHORDC1, XPNPEB2, LTA4H, RHOA, and CLDN1. (**C**) IPA network predicting reduced apoptosis in Asp4-CSE vs. CSE, involving B2M, YBX1, CAPNS1, DDX21, RAD23B, FUBP1, and MCAM. (**D**) IPA network predicting increased angiogenesis in Asp4-CSE vs. CSE, involving ANXA3, CAPN1, CAV1, SERPINC1, RHOA, MMP14, MYOF, and NF1. (**E**) Hierarchical clustering heatmap and pathway enrichment for Asp4-CSE vs. CSE. Blue arrow indicates angiogenesis enrichment. (**F**) IPA network predicting reduced apoptosis in Asp40-CSE vs. CSE. (**G**) Angiogenesis prediction for Asp40-CSE vs. CSE: No significant angiogenesis network is identified. (**H**) Heatmap and pathway enrichment for Asp40-CSE vs. CSE, with angiogenesis and apoptosis enrichment highlighted (red boxes). Each volcano plot point represents a protein plotted by log_2_ expression ratio (*x*-axis) and −log_10_-adjusted *p*-value (*y*-axis). In IPA networks, red and green nodes indicate increased and decreased protein measurements, respectively. Orange and blue indicate predicted activation and inhibition, with stronger color intensity reflecting higher confidence. Solid lines represent direct relationships and dashed lines represent indirect relationships. Orange, blue, yellow, relationship lines denote predicted activation, predicted inhibition, hypotheisized with the predicted state respectively.

Under prophylactic conditions, the CTC-derived EVs exhibited moderate recovery of angiogenic and structural remodeling pathways. The volcano plot analysis revealed distinct proteomic reprogramming (Figure 4A), with upregulation of SLC4A7, LGR4 (Wnt signaling receptor; endothelial proliferation), GPRC5C, IL17RA (immune modulation), THY1/CD90 (angiogenesis), and RHBIDF2. The IPA predicted reduced apoptosis (Figure 4C), with a network involving B2M, YBX1, CAPNS1, RPS27A, DDX21, RAD23B, FUBP1, and MCAM. Critically, the IPA simultaneously predicted increased angiogenesis (Figure 4D), with a network incorporating ANXA3, CAPN1, CAV1 (caveolin-1; VEGF signaling scaffold), COLTA1, SERPINC1, SARS1, RHOA, MMP14 (MT1-MMP; matrix remodeling), MYOF (myoferlin; VEGFR2 signaling), and NF1.

**Figure 5:**
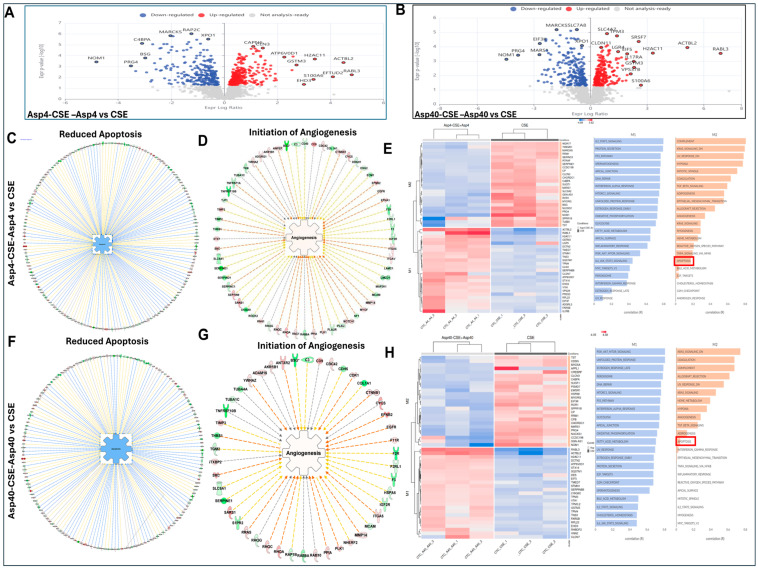
Low-dose prophylactic aspirin initiated to restore angiogenic signaling suppressed by oxidative injury. (**A**,**B**) Volcano plots depict differential gene expression profiles under prophylactic aspirin treatment paradigms in CTCs exposed to CSE. (**C**–**E**) Asp4-CSE-Asp4 vs. CSE: Low-dose aspirin pre-treatment reverses CSE-induced suppression, upregulating SLC4A7, LGR4, and RHOJ, and activating pathways associated with endothelial migration and extracellular matrix remodeling, consistent with enhanced angiogenesis. Red and green nodes indicate increased and decreased protein abundance, respectively, with color intensity reflecting the magnitude of change. Orange and blue relationship lines indicate predicted activation or inhibition effects, yellow lines indicate findings inconsistent with the predicted downstream state, and gray lines indicate relationships for which the effect is not predicted. Dashed lines represent indirect relationships, whereas solid lines represent direct relationships (**E**–**H**) Asp40-CSE-Asp40 vs. CSE: IPA causal network showing attenuation of CSE-induced apoptosis signaling. High-dose aspirin exerts minimal or neutral effects on angiogenic pathways, with only modest transcriptional changes and absence of significant enrichment in angiogenesis-related gene sets. Volcano plots show differential expression (log_2_ fold change vs. –log_10_ *p*-value); heatmaps and pathway enrichment plots illustrate relative gene expression and pathway correlation across treatment conditions. Blue indicates downregulated proteins/pathways, whereas red indicates upregulated proteins/pathways. Directional arrows represent predicted activation or suppression trends. Red boxes highlight key enriched biological processes/pathways.

The Asp4-CSE-Asp4 (prophylaxis exposure treatment) enhanced expression of VEGFA (log_2_ FC +0.7), COL4A1 (log_2_ FC +0.8, *p* = 0.004), and MMP14 (*p* = 0.047), suggesting partial restoration of endothelial signaling and extracellular matrix (ECM) organization, indicative of the membrane remodeling process after oxidative stress-induced damage (Figure 5A,C–E). The functional enrichment analysis confirmed the activation of VEGF, integrin, and cholesterol metabolism pathways after the prophylactic Asp treatment.

**High-Dose Co-Treatment (Asp40-CSE vs. CSE):** High-dose co-treatment produced fewer significant changes (Figure 4B), with modest alterations in CHORDC1, XPNPEB2, LTA4H, SRSF4, RHOA, and CLDN1. The IPA predicted reduced apoptosis (Figure 4F); however, no significant angiogenesis network was identified at this dose (Figure 4G). The pathway enrichment showed a distinct profile, with coagulation-related pathway enrichment (Figure 4H).

Low-dose prophylactic aspirin achieves dual rescue (anti-apoptotic + pro-angiogenic), while high-dose prophylaxis prevents apoptosis but fails to restore angiogenic signaling. This dose-dependent dissociation is a principal finding of the study (summarized in Table 4).

### 3.5. Continuous Low-Dose Prophylactic Aspirin Initiates Angiogenic Signaling Suppressed by Oxidative Injury

To evaluate whether extending aspirin exposure (before AND after CSE) provides additional benefit, we analyzed the continuous treatment groups Asp4-CSE-Asp4 and Asp40-CSE-Asp40 compared with CSE alone (Figure 5).

**Low dose, continuous (Asp4-CSE-Asp4 vs. CSE):** Extended low-dose aspirin exposure produced reprogramming (Figure 5A), with upregulation of MARCKS (membrane dynamics), RAP2C, CAPNS3, ATPV0D1 (vacuolar ATPase; EV biogenesis), H2AC11, GSTM3 (glutathione S-transferase; antioxidant defense), S100A6, EFTUD2, and RABL3 (vesicular trafficking). The IPA predicted both reduced apoptosis (Figure 5C) and initiation of angiogenesis (Figure 5D). The pathway enrichment confirmed restoration of angiogenesis-related signaling (Figure 5E, blue arrows).

**High dose, continuous (Asp40-CSE-Asp40 vs. CSE):** Unlike the high-dose co-treatment alone, the combined high-dose condition did show initiation of angiogenesis (Figure 5G), alongside reduced apoptosis (Figure 5E,F). The pathway enrichment showed TGF-β and angiogenesis-related pathway restoration (Figure 5G,H).

This demonstrates that extending aspirin exposure (before and after CSE) partially compensates for the dose-dependent limitation, with the most restoration in the low-dose combined group (summarized in Table 5).

### 3.6. Prophylactic Aspirin Suppresses Apoptosis and Inflammation in CTC-EVs

**Figure 6:**
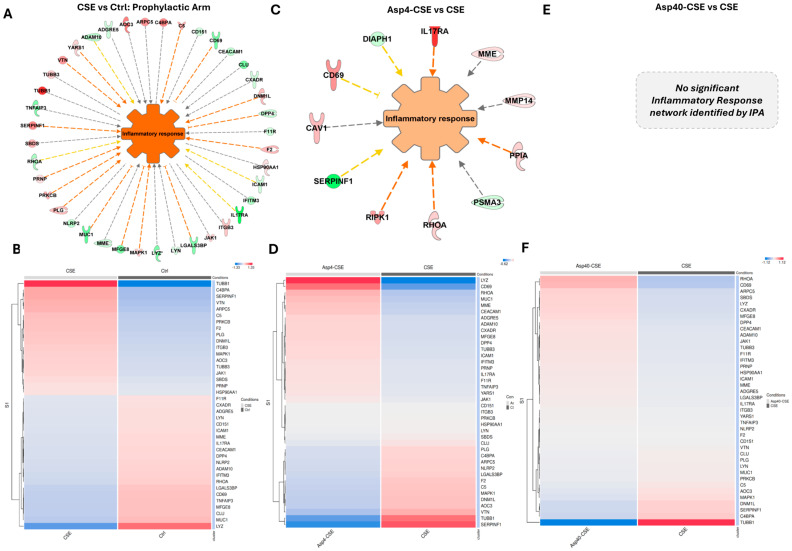
Timing and dose constrain aspirin-mediated modulation of inflammatory transcriptional networks. (**A**) IPA inflammatory response network: CSE vs. control. CSE activates a broad inflammatory network involving C5, CXADR, TNF, JAK1, ITGB3, and multiple adhesion molecules. (**B**) Hierarchical heatmap of inflammation-associated EV proteins: CSE vs. control. (**C**) IPA inflammatory response network: Asp4-CSE vs. CSE. Low-dose prophylactic aspirin produces a targeted network involving IL17RA, CD69, DIAPH1, CAV1, MMP14, SERPINF1, RIPK1, RHOA, and PPIA. (**D**) Heatmap of Asp4-CSE vs. CSE, showing normalization of inflammatory gene expression. (**E**) IPA inflammatory network for Asp40-CSE vs. CSE shows no significantly enriched inflammatory response network. (**F**) Heatmap of Asp40-CSE vs. CSE, showing minimal transcriptional reprogramming. Inflammatory modulation is exclusive to low-dose prophylactic aspirin and is absent at high dose.

To determine whether prophylactic aspirin modulates CSE-induced inflammation, an IPA network analysis was performed specifically for the inflammatory response pathways (Figure 6).

**CSE vs. Control** (Figure 6A,B): The CSE activated a broad inflammatory signaling network involving C5, CKADR, FKBP1A, C10TNF3, TRIM25, TNF, THBS4, THBS1, PLAU, LGALS3BP, JAK1, ITGB3, IGKC, IGHG1, HSPG2, and GCLM, with coordinated upregulation of adhesion molecules (ICAM1, CEACAM1, and CD151), proteases (ADAM10, DPP4, and MME), signaling kinases (LYN, MAPK1, and JAK1), and inflammatory mediators. The hierarchical clustering confirmed clear separation of CSE and control samples across inflammation-associated genes (Figure 6B), with proteins including TUBB1, C4BPA, SERPINF1, VTN, ARPC5, and MFG-E8 among the most differentially expressed.

**Asp4-CSE vs. CSE** (Figure 6C,D): Low-dose prophylactic aspirin produced a targeted inflammatory modulation network centered on the inflammatory response node, involving IL17RA (upregulated), DIAPH1, CD69 (upregulated), MME, CAV1, MMP14, SERPINF1 (downregulated), RIPK1, RHOA, PPIA, and PSMA3. The heatmap (Figure 6D) showed a clear shift toward normalization of CSE-induced inflammatory gene expression, with notable restoration of MFG-E8 expression and modulation of CEACAM1, ADAM10, and ICAM1.

**Asp40-CSE vs. CSE** (Figure 6E,F): In striking contrast, the high-dose prophylactic aspirin did not yield a statistically enriched inflammatory response network in the IPA, indicating an absence of coordinated transcriptional modulation of inflammatory mediators at this dose. The heatmap (Figure 6F) showed minimal transcriptional reprogramming compared with the low-dose condition.

This finding demonstrates that inflammatory modulation by prophylactic aspirin is exclusive to a low dose and is absent at high doses, paralleling the angiogenesis dose dependency.

### 3.7. Continuous Aspirin Followed by CSE-Induced Oxidative Stress Attenuates Inflammation and Restores Metabolic Homeostasis

**Figure 7:**
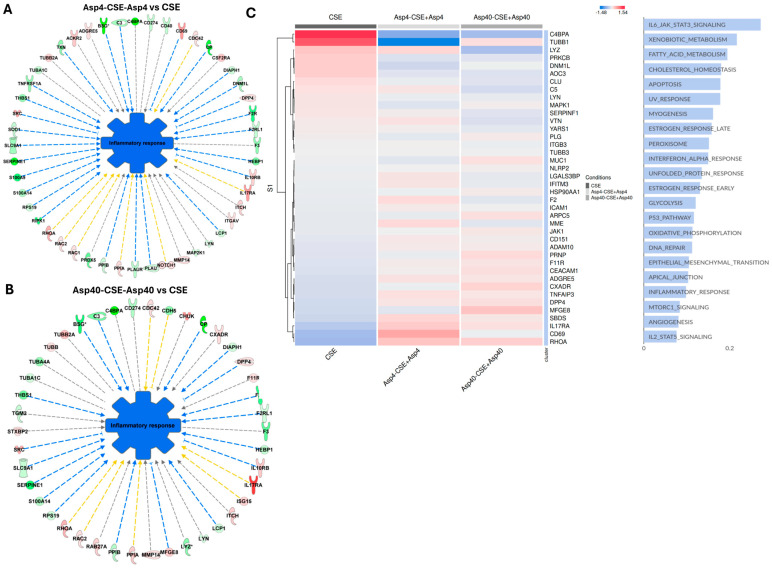
Aspirin continuation following oxidative stress selectively attenuates inflammatory signaling and restores metabolic homeostasis. (**A**) IPA inflammatory response network: Asp4-CSE-Asp4 vs. CSE. Coordinated downregulation (green nodes) of inflammatory mediators with mixed activation/inhibition relationships. (**B**) IPA inflammatory network: Asp40-CSE-Asp40 vs. CSE. Broader but less directional transcriptional response. (**C**) Hierarchical heatmap of inflammation-associated proteins across CSE, Asp4-CSE-Asp4, and Asp40-CSE-Asp40 conditions. Asp4-CSE-Asp4 shows the most consistent normalization. Key proteins: C4BPA, TUBB1, LYZ, PRKCB, CLU, MAPK1, SERPINF1, VTN, MFG-E8, ICAM1, CD151, CD69, and RHOA. S1 module pathway correlations showing aspirin continuation influences IL-6/JAK-STAT signaling, xenobiotic metabolism, cholesterol homeostasis, oxidative phosphorylation, DNA repair, apical junction, inflammatory response, angiogenesis, and mTORC1 signaling. Node color: red = upregulated; green = downregulated. Edge color: orange = predicted activation; blue = predicted inhibition. Blue indicates downregulated proteins/pathways, whereas red indicates upregulated proteins/pathways. Directional arrows represent predicted activation or suppression trends. Red boxes highlight key enriched biological processes/pathways.

To assess whether extending aspirin after CSE exposure enhances inflammatory resolution, combined treatment groups were analyzed for their inflammatory networks (Figure 7).

Asp4-CSE-Asp4 vs. CSE (Figure 7A): The IPA revealed a large, coordinated network showing predominant downregulation (green nodes) of inflammatory mediators, with both activation (orange edges) and inhibition (blue edges) relationships, indicating active remodeling of the inflammatory landscape. The network included CXADR, DPP4, F2K, F11R, CD274, CXCL2, ADAM10, and numerous downstream targets.

Asp40-CSE-Asp40 vs. CSE (Figure 7B): The high-dose combined condition also showed inflammatory network modulation, though with a broader but less directional pattern, featuring both up- and downregulated nodes.

Heatmap comparison (Figure 7C): Hierarchical clustering of inflammation-associated proteins across CSE, Asp4-CSE-Asp4, and Asp40-CSE-Asp40 conditions demonstrated that Asp4-CSE-Asp4 showed the most consistent normalization toward control-like expression, while Asp40-CSE-Asp40 showed partial modulation. The key normalized proteins included C4BPA, TUBB1, LYZ, PRKCB, DMKL1, AOC3, CLU, LYN, C5, MAPK1, SERPINF1, VTN, and MFG-E8.

Pathway correlations (Figure 7C): The S1 module pathway analysis revealed that continuous aspirin treatment influenced IL-6/JAK-STAT signaling, xenobiotic metabolism, fatty acid metabolism, cholesterol homeostasis, oxidative phosphorylation, DNA repair, apical junction integrity, inflammatory response, angiogenesis, and mTORC1 signaling.

These data indicate that continued low-dose aspirin following oxidative insult preferentially suppresses inflammatory signaling while simultaneously promoting metabolic and epithelial homeostasis.

### 3.8. EVs Encode Partial Attenuation of Apoptotic and Necrotic Signaling Following Post-Injury Therapeutic Aspirin Exposure

**Figure 8:**
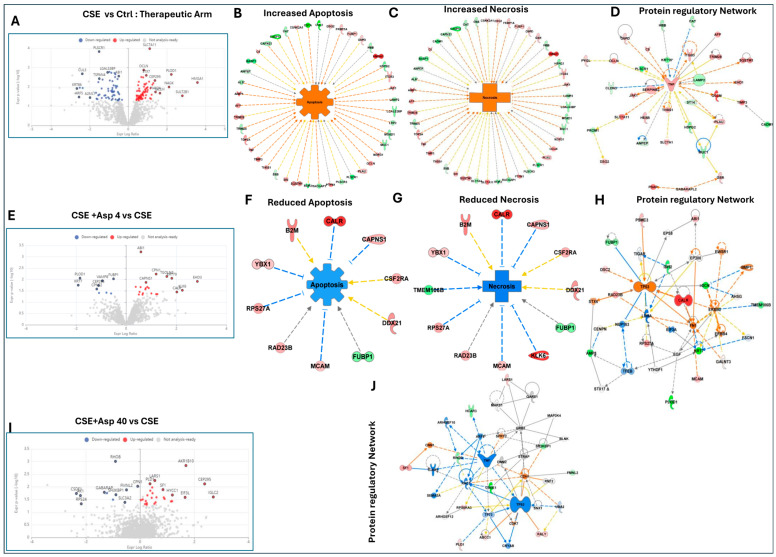
Therapeutic aspirin attenuates oxidative stress-induced apoptotic and necrotic signaling in CTC-EVs. (**A**) Volcano plot: CSE vs. control (therapeutic arm). Upregulated: SLC7A11, OGLN, CEP205, NAGK, HMAGA1, and SULTZ81. Downregulated: PLSCR1, GUL8, LGALS3BP, TSPAN6, and KRT66. (**B**,**C**) IPA networks predicting increased apoptosis (**B**) and increased necrosis (**C**) in CSE vs. control. (**D**) Protein regulatory network: CSE vs. control. (**E**) Volcano plot: CSE+Asp4 vs. CSE. Fewer significant changes; notable: AB1, PLOD1, CAPNS1, and EHQ2. (**F**,**G**) IPA networks predicting reduced apoptosis (**F**) and reduced necrosis (**G**) in CSE+Asp4 vs. CSE. Networks involve CAPNS1, YBX1, CSF2RA, DDX21, RPS27A/K, RAD23B, FUBP1, and MCAM. (**H**) Protein regulatory network: CSE+Asp4 vs. CSE. (**I**) Volcano plot: CSE+Asp40 vs. CSE. Broader responses include RHO5, AKR1B10, CEP295, SF1, and IGLC2. (**J**) Protein regulatory network for CSE+Asp40 vs. CSE: Shows extensive remodeling.

**CSE Damage in the Therapeutic Arm (CSE vs. Control;** Figure 8A–D**):** In the therapeutic experimental arm, CSE exposure (designated CSE 4, reflecting the therapeutic protocol timing) induced significant proteomic changes, with upregulation of SLCAT11, OGLN, and stress-responsive proteins (partial stress adaptation), and downregulation of PLSCR1, GUL8, LGALS3BP, TSPAN6, and KRT66. The IPA predicted increased apoptosis (Figure 8B), increased necrosis (Figure 8C), and activated protein regulatory networks (Figure 8D same as Supplementary Figure S1A,B). This indicates an EV-encoded shift away from pro-apoptotic network states rather than complete suppression of death signaling.

**Low-Dose Therapeutic Aspirin (CSE+Asp4 vs. CSE;** Figure 8E–H**)**

Therapeutic low-dose aspirin produced fewer differentially expressed proteins than the prophylactic conditions (Figure 8E), with modest changes in AB1, PLOD1, VAVHE8, CAPNS1, IGOLM75, EHQ2, and CALA55. Despite limited individual protein changes, the IPA predicted reduced apoptosis (Figure 8F), with a network involving GAPR, CAPNS1, YBX1, CSF2RA, DDX21, RPS27K, RAD23B, FUBP1, and MCAM, and reduced necrosis (Figure 8G), with a parallel network including B2K, CAPNS1, YBX1, TMEM198B, CSF2RA, DDX21, RPS27A, RAD23B, FUBP1, and MCAM. The EVs generated under low-dose therapeutic aspirin exposure also displayed reduced enrichment of inflammatory and necrosis-associated pathways (Figure 8E–H, Supplementary Figure S3A,B).

**High-Dose Therapeutic Aspirin (CSE+Asp40 vs. CSE;** Figure 8I,J**)**

High-dose therapeutic aspirin (Figure 8I) produced a broader transcriptional response than the low dose, with upregulation of RHO5, AKR1B10, CEP295, SF1, IGLC2, and others. The protein regulatory network (Figure 8J) demonstrated extensive remodeling, with both activation and inhibition interactions. In contrast to the low dose, the EV cargo from the high-dose aspirin conditions showed decreased representation of survival-associated proteins, including MCL1- and AKT1-linked networks, suggesting that excessive pharmacologic inhibition may constrain EV-encoded adaptive recovery programs (Figure 8I,J). Network-level analyses of EV proteomic states demonstrated (Supplementary Figure S1A,B) that low-dose therapeutic aspirin most effectively limited EV-encoded apoptotic signaling while preserving reparative programs (Supplementary Figure S3A,B), whereas high-dose exposure (Supplementary Figure S3C,D) broadly suppressed both damage and repair-associated EV networks, consistent with a dormant or constrained adaptive state. Both therapeutic doses attenuated CSE-induced apoptosis and necrosis, but through networks of modest individual protein changes, suggesting that therapeutic aspirin achieves partial rescue through subtle, distributed effects rather than the reprogramming seen with prophylactic treatment.

### 3.9. Therapeutic Aspirin Fails to Reverse CSE-Induced Inflammatory Programming and Timing-Dependent Prophylactic Effects Reveal Limited Capacity of Aspirin to Reprogram CTC Inflammation

**Figure 9:**
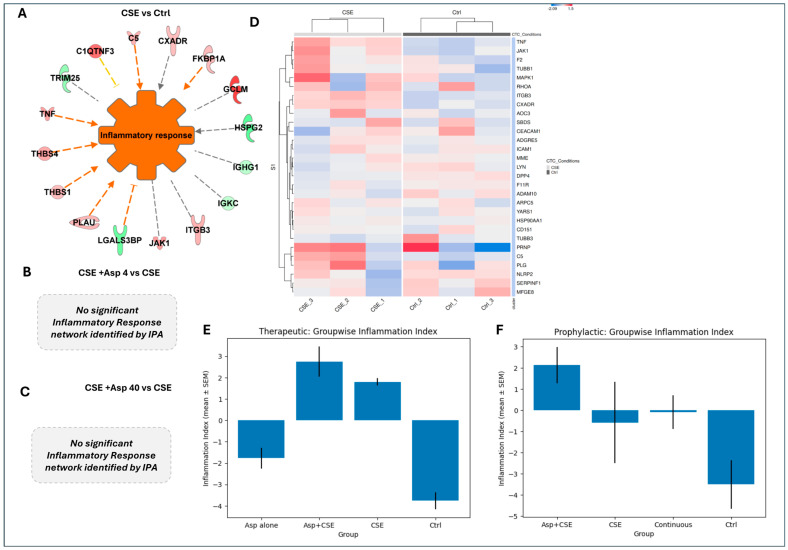
CSE induces a strong inflammatory transcriptional program that is not reversed by therapeutic aspirin and the composite Inflammation Index reveals that only continuous aspirin normalizes inflammatory signaling. (**A**) IPA inflammatory response network: CSE vs. control. CSE induces coordinated upregulation of inflammatory mediators, including C5, CKADR, FKBP1A, GCLM, HSPG2, IGHG1, TRIM25, TNF, THBS4/THBS1, PLAU, JAK1, and ITGB3. Red and green nodes indicate increased and decreased protein abundance, respectively, with color intensity reflecting the magnitude of change. Orange and blue relationship lines indicate predicted activation or inhibition effects, yellow lines indicate findings inconsistent with the predicted downstream state, and gray lines indicate relationships for which the effect is not predicted. Dashed lines represent indirect relationships, whereas solid lines represent direct relationships (**B**) CSE+Asp4 vs. CSE shows no significantly enriched inflammatory response network in the IPA. Therapeutic low-dose aspirin does not yield a significantly enriched inflammatory response network in the IPA. (**C**) CSE+Asp40 vs. CSE similarly shows no enriched inflammatory response network. (**D**) Hierarchical heatmap of inflammation-associated proteins: CSE vs. control. The CSE samples cluster distinctly from the controls, with consistent induction of TNF, JAK1, F2, TUBB1, MAPK1, RHOA, ITGB3, and CXADR, and suppression of PRNP, TUBS3, NLRP2, SERPINF1, and MFG-E8. Together, these data demonstrate that therapeutic aspirin is insufficient to reverse CSE-established inflammatory transcriptional states at either dose, highlighting the critical importance of aspirin timing in immunomodulation at the feto-maternal interface. (**E**) Therapeutic arm: The groupwise Inflammation Index is calculated as z(IL-6) + z(IL-8) + z(TNF) − z(IL-10). Aspirin alone shows low inflammation, comparable to that of control (Ctrl). CSE elevates the index. Therapeutic aspirin following CSE (Asp+CSE) does not reduce the index and trends higher than CSE alone, indicating failure to resolve and potentially exacerbating inflammatory signaling. (**F**) Prophylactic arm: Asp+CSE (co-treatment) shows an elevated Inflammation Index higher than CSE alone. However, the continuous schedule (Asp-CSE-Asp; red box) preserves the Inflammation Index near the baseline, with values comparable to the control. CSE alone shows a modest positive index. The data represent mean ± SEM of n = 3 biological replicates. These data demonstrate that only continuous aspirin exposure (before and after CSE) achieves normalization of the inflammatory milieu; neither the single-phase prophylactic nor therapeutic aspirin is sufficient. Blue indicates downregulated proteins/pathways, whereas red indicates upregulated proteins/pathways. Directional arrows represent predicted activation or suppression trends. Red boxes highlight key enriched biological processes/pathways.

Exposure to CSE elicited a strong inflammatory response in the CTCs, marked by pronounced increases in IL-6 and IL-8 secretion, minimal alteration in TNF, and a modest decline in IL-10 supported by the IPA network analysis (Figure 9A–D). In the therapeutic treatment paradigm, aspirin did not mitigate this CSE-driven activation. The cytokine levels in the CSE+Asp4 and CSE+Asp40 groups remained similar to, or in some cases exceeded, those observed with CSE alone. No networks were produced by the IPA. In case of treatment (Figure 9B,C), TNF production was largely unaffected. Notably, the IL-10 concentrations were further reduced by aspirin, leading to elevated IL-6/IL-10 and TNF/IL-10 ratios. Consistent with these patterns, the composite z-scored Inflammation Index (IL-6 + IL-8 + TNF − IL-10) reached its highest value in the CSE+Asp40 condition, indicating that therapeutic aspirin failed to resolve and may have even intensified the inflammatory imbalance in the CTCs shown in Figure 9A. In the prophylactic paradigm, similar resistance to aspirin was observed. Co-treatment with Asp4 or Asp40 failed to prevent CSE-induced IL-6 and IL-8 upregulation, and these groups displayed higher IL-6/IL-10 ratios than the CSE alone. Only the continuous exposure schedule (Asp → CSE → Asp) partially restored IL-10 and produced the modest reduction in the Inflammation Index shown in Figure 9D,E, suggesting that transient prophylactic dosing is insufficient to reprogram CTC inflammatory output.

### 3.10. Comparative Analysis Across All Treatment Paradigms

Integrating the findings from all prophylactic, therapeutic and continuous treatments, a hierarchy of angiogenic rescue emerges, which is summarized in Table 6. The critical finding is that enrichment of EV-encoded angiogenic cargo, as predicted by the IPA pathway analysis, was preferentially observed with low-dose aspirin, with the most distinct enrichment occurring in the Asp4-CSE co-treatment and Asp4-CSE-Asp4 combined conditions. The high-dose aspirin co-treatment (Asp40-CSE) did not show enrichment of the predicted angiogenic EV signature, while the extended high-dose exposure (Asp40-CSE-Asp40) showed only partial enrichment. We emphasize that these are EV cargo and pathway-prediction signatures and not direct measures of in situ angiogenic activity, which would require functional vascular assays for confirmation. The pro-angiogenic program restored by low-dose aspirin involves a coordinated set of proteins with established roles in vascular development: the key protein drivers are listed in Table 7.

### 3.11. Machine Learning Algorithm Identifies Distinct Prophylactic and Therapeutic EV Biomarker Signatures

To identify key molecular predictors of aspirin responsiveness, we employed a machine learning (ML)-driven feature selection workflow using the BigOmics platform across the prophylactic and therapeutic CTC treatment groups. Several independent ML algorithms under platform [38], which includes sPLS, elastic nets, random forests, and extreme gradient boosting, converged on a shared panel of highly discriminative features identified candidate biomarkers features across CSE, Asp4-CSE, and Asp4-CSE–Asp4 conditions (Figure 10A).

The integrated heatmap derived from the ML models revealed consistent clustering of prophylactic aspirin-treated CTCs (Asp4-CSE-Asp4 and Asp40-CSE-Asp40) apart from the oxidative stress and control groups, reflecting a coordinated reprogramming of the CTC proteome. Among the top-ranked features were RPS23, TUBA4A, RTCB, SPRR2E, HSPA8, HNRNPU, SLC1A3, and SERPINF2, which were also independently enriched in the IPA biomarker analyses of EV cargo associated with angiogenesis, cytoskeletal integrity, and oxidative stress recovery pathways (Figure 10B).

The bar plot validation from the proteomic dataset confirmed differential abundance of these candidates in the prophylactic aspirin conditions relative to the CSE-treated controls. Notably, SERPINF2 (α2-antiplasmin) and HSPA8 (heat shock cognate 71 kDa protein) were significantly upregulated during prophylactic aspirin exposure, indicating reinforcement of anti-apoptotic and stress resilience networks. At the same time, RPS23 and TUBA4A suggested recovery of translational and cytoskeletal stability.

To define the hierarchical decision logic underlying group separation, a classification tree was constructed using the top-ranked proteomic features (Figure 10C). RPS23 (≥18) emerged as the primary discriminating node, separating the four aspirin-exposed conditions (67% of samples) from the CSE and Ctrl (33%). On the aspirin branch, sequential splits at TUBA4A (≥19), RTCB (≥17), and SPRR2E (<16) progressively resolved Asp4-CSE, Asp4-CSE+Asp4, Asp40-CSE, and Asp40-CSE+Asp40 into distinct terminal nodes; on the non-aspirin branch, a single split at TUBA4A (≥20) cleanly separated CSE from the Ctrl. This compact decision rule, built on just four features, was sufficient to recapitulate the global clustering observed in the heatmap, supporting their robustness as classifiers of aspirin-mediated protection.

To assess the stability of these candidates across independent feature selection algorithms, the cumulative feature importance ranks were aggregated from eight ML methods, spanning multivariate (sparse PLS-DA, random forest, XGBoost with linear and tree boosters, and elastic net regularized regression [glmnet]) and univariate filters (F-test and correlation) (Figure 10D). SPRR2E, HSPA8, TUBA4A, RAB6A, SERPINF2, HNRNPU, SLC1A3, and RPS23 consistently emerged among the highest-ranked proteins across all eight methods, confirming that their predictive value is not method-dependent but reflects a genuine biological signal within the EV proteome.

Collectively, the combination of ML-driven feature discovery and proteomic cross-validation establishes a distinct biomarker panel predictive of aspirin-mediated protection in chorion trophoblasts. These markers represent measurable candidates for translational EV-based diagnostic development in maternal plasma.

### 3.12. AI/ML-Driven Therapeutic Biomarker Identification and Proteomic Validation

The machine learning analysis of therapeutic conditions (CSE → Asp4 and CSE → Asp40) revealed distinct proteomic signatures that reflect dose-dependent aspirin responses. The ML-integrated heatmap (Figure 11A) shows CSE → Asp4 clustering toward partial recovery and CSE → Asp40 forming a separate cluster characterized by a more restrictive, stress-adapted profile. The features contributing most strongly to this separation include COL4A1, SLC4A1, EIF3L, EDNRB, KARS1, and CLDN7 in the CSE → Asp4 group, indicating restoration of ECM organization, translational activity, and epithelial barrier integrity. Conversely, IGLC2, ACO2, PRSS3P2, TXNDC17, and RAB1B are enriched in the CSE→Asp40 group, consistent with metabolic suppression, redox regulation, and altered proteostasis under high-dose aspirin.

The proteomic boxplot validation (Figure 11B) confirmed these ML-derived distinctions. CEP295, SERPINB4, PLOD1, PVR, and EHD3 were elevated primarily in CSE → Asp40, aligning with the heatmap-defined stress adaptation module. In contrast, KRT79, RHOB, and RAB23 showed greater recovery in CSE → Asp4, supporting the ML prediction that low-dose therapeutic aspirin promotes partial restoration of epithelial organization and GTPase-mediated trafficking pathways. Together, the heatmap clustering and orthogonal proteomic validation establish a coherent set of therapeutic biomarkers demonstrating that CSE → Asp4 induces a recovery-oriented phenotype, whereas CSE → Asp40 drives a distinct metabolic and stress-adapted state.

To define the hierarchical decision logic underlying group separation, a classification tree was constructed using the top-ranked proteomic features (Figure 11C). KRT7 (≥17) emerged as the primary discriminating node, separating CSE+Asp4 from the remaining conditions; a subsequent split at SERPINB4 (≥17) cleanly resolved CSE from CSE+Asp40. This compact two-feature decision rule was sufficient to reconstruct the global clustering pattern observed in the heatmap, supporting KRT7 and SERPINB4 as the most informative classifiers of therapeutic aspirin response.

To assess the stability of these candidates across independent feature selection algorithms, cumulative feature importance ranks were aggregated from eight ML methods, spanning multivariate (sparse PLS-DA, random forest, XGBoost with linear and tree boosters, and elastic net regularized regression [glmnet]) and univariate filters (F-test and correlation) (Figure 11D). KRT7, SERPINB4, PLOD1, and PVR consistently emerged among the highest-ranked proteins across all eight methods, confirming that their predictive value is not method dependent but reflects a genuine biological signal within the EV proteome.

These integrated findings further highlight the utility of AI/ML approaches for defining aspirin-responsive molecular signatures in CTCs, which might be useful for clinical decision making.

## 4. Discussion

This study characterizes, for the first time, the dose- and timing-dependent effects of aspirin on CTCs and their corresponding extracellular vesicle (EV) cargo under oxidative stress conditions that mimic preeclampsia (PE) pathology. CTCs, located at the feto-maternal decidua interface, are known to function as a mechanical barrier, an immunoregulatory layer, and a major source of the pregnancy hormone progesterone. CTC-derived EVs represent key paracrine mediators within the amniochorionic microenvironment, capable of influencing inflammation, angiogenesis, and extracellular matrix (ECM) integrity [28,40,41]. Using EV proteomics as a molecular readout of cellular status, we demonstrate that low-dose aspirin modulates anti-apoptotic and anti-inflammatory pathways in CTCs in a time- and dose-dependent manner, extending the current understanding of aspirin’s action beyond the placenta, highlighting a secondary but physiologically relevant pharmacological effect on the fetal membrane interface.

### 4.1. CSE Creates a “Pathological EV Signature” of Coagulation, Apoptosis, and Vascular Suppression: F10 and MFG-E8 as Anchors of a Pathological Program

The marked upregulation of F10 (Coagulation Factor X) as the single most significantly altered protein in the CSE-derived EVs might indicate a direct mechanistic link between CSE exposure and the prothrombotic state characteristic of placental pathology. EV-associated coagulation factors contribute to the thrombo-inflammatory microenvironment of PE [42,43], and our findings suggest that the CTC-EVs from smoking-affected pregnancies actively promote coagulation at the feto-maternal interface. Notably, F10 is detectable by standard clinical coagulation assays, positioning it as an immediately translatable biomarker candidate. The downregulation of MFG-E8 (Lactadherin) may suggest multiple implications. MFG-E8 is known to promote efferocytosis, inhibit inflammation, and support angiogenesis through αvβ3/αvβ5 integrin engagement [44]. On EVs, MFG-E8 facilitates vesicle—target cell interactions. Its depletion from CSE-derived EVs simultaneously impairs anti-inflammatory clearance, pro-angiogenic signaling, and EV uptake efficiency, resulting in a compounded loss of protective functions.

The downregulation of VPS4B, an ESCRT AAA-ATPase essential for EV biogenesis [45], parallels the identification of BROX (also ESCRT associated) in the ML panel (Figure 11) and suggests that CSE disrupts the EV packaging machinery itself. This disruption may explain the coordinated, multi-pathway perturbation observed across the CSE-derived EV proteome.

The coordinate activation of TNFα/NF-κB, IL-6/JAK/STAT3, p53, interferon-γ, hypoxia, and inflammatory response pathways in the CSE-derived EV cargo (Figure 3D) mirrors the molecular landscape of a preeclamptic placenta [46,47,48], suggesting that CTC-EVs from CSE-exposed cells carry a molecular program that, if transferred to maternal cells, could contribute to the systemic inflammatory and endothelial activation characteristic of PE.

### 4.2. Aspirin Demonstrates a Compartment-Specific Molecular Response Within the Feto-Maternal Interface

While aspirin’s vascular and anti-inflammatory benefits in the placenta are well documented, its impact on the CTC layer, a critical yet distinct component of the fetal membrane complex, has remained uncharacterized. Our proteomics analysis reveals a fundamental functional compartmentalization at the feto-maternal interface. Previous studies have characterized the placenta as the primary pharmacological target, responding to aspirin with angiogenic reprogramming. In contrast, we identify the CTC layer as a distinct ‘Secondary Responder.’ Unlike placental trophoblasts, which drive vascular remodeling, CTCs respond to prophylactic aspirin by reinforcing structural and survival checkpoints. This suggests that aspirin’s clinical efficacy in preventing preeclampsia is not solely derived from placental perfusion, but also from the stabilization of the chorio-decidual barrier, potentially mitigating the ‘second hit’ of inflammatory cascade that precipitates preterm birth. This compartmentalized response aligns with the physiological roles of these tissues. The placenta mediates nutrient and gas exchange, demanding distinct angiogenic control, while the chorion primarily maintains intrauterine structural integrity, endocrine functions and immune tolerance. Aspirin’s modest but measurable effects in CTCs may thus serve to maintain immune equilibrium and prevent premature inflammatory activation, a plausible mechanism contributing to its observed reduction in preterm birth risk in clinical settings.

### 4.3. Dose- and Timing-Dependent Dissociation: A Pharmacological Window for Angiogenic Rescue

The finding that only low-dose aspirin restores angiogenesis, while both doses prevent apoptosis, represents the most clinically significant observation of this study, revealing a clear dose-dependent dissociation in the CTC-EV responses. Low-dose aspirin selectively inhibits COX-1-mediated thromboxane A_2_ while sparing COX-2-dependent prostacyclin, thereby preserving PGI_2_-driven trophoblast invasion, vascular remodeling, and angiogenesis [49,50], which may sustain pro-angiogenic signaling (LGR4, THY1, CAV1, MMP14, and MYOF). In contrast, high-dose aspirin inhibits both COX-1 and COX-2, suppressing PGI_2_ as well as TxA_2_ [51]. The loss of PGI_2_ may negate the pro-angiogenic benefits despite the anti-inflammatory effects, while the residual anti-apoptotic activity likely reflects COX-independent mechanisms (e.g., NF-κB inhibition, antioxidant actions). Low-dose prophylactic aspirin restores a coordinated angiogenic program comprising LGR4 (Wnt/β-catenin activation), THY1/CD90 (adhesion-mediated angiogenesis), CAV1 (VEGFR scaffolding), MMP14/MT1-MMP (ECM remodeling for invasion), and MYOF (VEGFR2 trafficking/signaling), supporting endothelial growth [52,53,54,55,56,57,58,59]. When aspirin was administered prophylactically (Asp4-CSE-Asp4) before oxidative stress, the CTC-derived EVs showed early suppression of apoptotic mediators (CASP3, BAX) and restoration of ECM-related and angiogenic proteins (MMP14, COL4A1). This pattern suggests that prophylactic aspirin preconditions the chorion, establishing a low-inflammatory and pro-cell survival baseline that resists oxidative insult. In contrast, therapeutic aspirin (CSE-Asp4) administered post-injury acted primarily as a rescue mechanism, reducing TNF and p53 activation and stabilizing EV-mediated stress signaling. The ability of post-injury aspirin to restore molecular homeostasis indicates that the drug retains reparative potential even when the prophylactic window has been missed. However, the effects were more limited in scope, consistent with clinical data showing reduced efficacy when aspirin is initiated late in gestation (>16 weeks). High-dose aspirin in both paradigms suppressed not only injury but also adaptive responses, including repair and angiogenic programs, a pattern indicative of over-inhibition of eicosanoid and NF-κB pathways, reinforcing the concept of a narrow therapeutic window.

### 4.4. The Inflammatory Divide: Prophylaxis Modulates, Therapy Cannot Reverse

Perhaps the most important clinical finding is the complete inability of therapeutic aspirin to reverse CSE-established inflammatory programming (Figure 9). At both therapeutic doses, the IPA failed to identify a significantly enriched inflammatory response network, while the heatmap confirmed persistent inflammatory gene activation indistinguishable from untreated CSE. This finding illuminates why aspirin initiated after 16 weeks of gestation has shown diminished efficacy in clinical trials [7,9]. By mid-pregnancy, CSE exposure has already established inflammatory programming in trophoblasts. Aspirin initiated at this point can modulate apoptosis and necrosis through COX-independent mechanisms, but cannot disassemble the entrenched inflammatory transcriptional state: it is, in effect, too late for the inflammatory component of protection. Conversely, prophylactic low-dose aspirin, particularly in the continuous schedule (Asp4-CSE-Asp4), modulates inflammatory networks (Figure 6 and Figure 7) and normalizes the composite Inflammation Index (Figure 9E). This observation suggests that aspirin must be present at the time of or before the inflammatory insult to prevent establishment of the inflammatory program, rather than attempting to reverse it afterward.

Notably, the prophylactic co-treatment (Asp+CSE) paradoxically increased the Inflammation Index (Figure 9F), whereas continuous treatment normalized it toward the baseline. One interpretation is that acute aspirin co-administration during CSE exposure transiently augments inflammatory signaling, but sustained exposure allows the anti-inflammatory program to establish and predominate. This carries direct clinical implications: consistent, continuous aspirin exposure may be more important than the specific timing of initiation [13,60].

### 4.5. The Therapeutic Arm: Partial Rescue Through Subtle Redistribution

While therapeutic aspirin cannot reverse established inflammatory programming, it does attenuate apoptosis and necrosis (Figure 8) through distributed, modest protein changes rather than the strong reprogramming observed with the prophylactic regimen. The therapeutic networks involve many of the same nodes as the prophylactic networks (CAPNS1, YBX1, DDX21, FUBP1, MCAM, and RAD23B), but with smaller effect sizes. The analysis in Supplementary Figure S3 reveals that therapeutic low-dose aspirin (CSE+Asp4) achieves partial rescue but with persistent predicted DNA damage and inflammatory outcomes, while therapeutic high-dose aspirin (CSE+Asp40) produces the most complex network, with evidence of both protective and adverse (DNA damage, inflammation of organ) outcomes. Collectively, these findings support a clinical model where therapeutic aspirin offers incomplete protection: sufficient to modulate selected pathological pathways but inadequate for comprehensive rescue, particularly of inflammatory and angiogenic domains.

### 4.6. EVs as Mechanistic Biomarkers of Aspirin Action

These findings provide a quantitative foundation for precision biomarker discovery in aspirin-mediated rescue, consistent with emerging EV-based biomarker frameworks in pregnancy [10,13,61]. By integrating machine learning with high-resolution proteomics, an approach increasingly used for biomarker discovery [62,63], we identified a molecular ‘signature of protection’ that defines the prophylactic window. The specific enrichment of SERPINF2 (α2-antiplasmin) and HSPA8 in the EVs of aspirin-protected cells serves as a functional readout of the protective state engendered by SERPINF2, a potent inhibitor of fibrinolysis and apoptosis, which likely acts as an ‘anti-apoptotic anchor’, preventing the necrotic drift observed in untreated/CSE-treated cells. The detection of these markers in CTC-derived EVs suggests their potential utility as non-invasive liquid biopsy candidates. Unlike non-specific inflammatory markers, such as CRP, a composite EV-derived ‘Aspirin Response Score’ could clinically differentiate between patients who are successfully buffered by prophylaxis and ‘non-responders’ who require alternative interventions. Because the PLAP^+^ EVs are fetal in origin, they are detectable in maternal circulation, and could therefore be used to monitor feto-placental membrane well-being using minimally invasive samples. This supports the translational concept that PLAP^+^ EVs or other chorion-enriched EV populations could serve as biomarkers of aspirin responsiveness. Unlike static tissue biopsies, EVs provide a real-time, non-invasive readout of drug efficacy and tissue adaptation. Such biomarkers could stratify pregnancies into aspirin responders versus non-responders, and inform clinical decisions regarding dosage and initiation timing.

### 4.7. Toward a Unified Model: Timing, Dose, and Duration

Integrating all the findings, we propose a three-tiered unified model of aspirin’s effects on CTC-EV cargo, stratified by their pharmacological accessibility. Apoptosis prevention, the most broadly accessible effect, was achieved by all aspirin regimens at both doses regardless of timing, likely reflecting COX-independent mechanisms, including NF-κB modulation, direct protein acetylation, and antioxidant activity. Angiogenic restoration was selectively achieved only by low-dose aspirin, particularly when administered before or during CSE exposure, consistent with preservation of COX-2-dependent prostacyclin signaling within the narrow pharmacological selectivity window afforded by low-dose aspirin. Inflammatory modulation was the most restricted effect, achievable only prophylactically at a low dose and requiring continuous exposure for full normalization of the Inflammation Index; once inflammatory programming was established, this effect was completely therapeutically inaccessible. This hierarchical model, in which anti-apoptotic protection is universally accessible, angiogenic rescue is dose-restricted, and inflammatory modulation is both dose- and timing-restricted, provides a mechanistic rationale for why early, continuous, low-dose aspirin affords the most comprehensive protection, consistent with the ASPRE trial [7] and ISSHP guidelines [64].

### 4.8. Clinical and Translational Relevance

Clinically, these findings underscore that aspirin’s therapeutic benefit may extend beyond its classical anti-platelet or placental actions. Even subtle modulation of fetal membrane signaling could contribute to improved pregnancy outcomes, particularly in reducing inflammation-driven complications, such as preterm premature rupture of membranes (PPROM) and spontaneous preterm birth. The dose- and timing-dependent relationships characterized here provide a mechanistic rationale for initiating low-dose aspirin early in pregnancy to maximize its protective efficacy while preserving regenerative and angiogenic processes. Furthermore, the identification of EV-based biomarkers that distinguish aspirin-responsive from aspirin non-responsive states opens new avenues for personalized obstetric treatment approaches. Monitoring EV proteomic profiles longitudinally during gestation might help to identify non-responders early, guiding timely dosage adjustments or adjunctive interventions.

### 4.9. Study Limitations

Several limitations should be considered when interpreting these findings. First, this is an entirely in vitro study performed using a single immortalized chorion trophoblast cell line derived from one uncomplicated term donor; the results therefore reflect the response of one genetic background and one cell type and do not capture inter-individual variability, donor-specific susceptibility, or the multicellular complexity of an intact feto-maternal interface. Second, oxidative stress was modeled using cigarette smoke extract (CSE) as a single, well-characterized inducer; while CSE recapitulates several molecular features reported for preeclamptic placentae, the etiology of PE is heterogeneous and likely involves additional contributors (hypoxia/reoxygenation, sFlt-1 imbalance, and ischemia–reperfusion) that this model does not address. Future work using microphysiological systems, organ-on-chip platforms, and primary CTCs from PE donors will be required to test the generalizability of the EV signatures reported here. Third, all the experiments used n = 3 biological replicates; this is sufficient for a hypothesis-generating exploration, but limits the statistical power, increases the variance in cytokine and inflammation index measurements, and constrains the generalizability of the machine learning biomarker panel. The ML analysis is therefore presented as an exploratory feature discovery rather than a clinically validated predictive model. Fourth, several mechanistic conclusions (predicted angiogenic recovery, predicted apoptosis suppression, and predicted inflammatory modulation) rest on Ingenuity Pathway Analysis-derived enrichment scores rather than on direct functional assays; key candidate effectors (e.g., VEGFA, MMP14, COL4A1, HSPA8) have not yet been validated by orthogonal techniques, such as Western blot, ELISA, or functional EV-uptake assays on recipient endothelial or trophoblast cells, and these confirmatory studies are planned as the immediate next step. Fifth, the candidate plasma biomarkers identified by ML (HSPA8, SERPINF2, COL4A1, and PLOD1) have not been validated in maternal plasma from PE patients or aspirin-responsive/non-responsive cohorts; their clinical utility therefore remains hypothetical until prospective patient validation is performed. Finally, an apparent discrepancy between the EV proteomic predictions of attenuated inflammation under low-dose prophylactic aspirin and the more limited cytokine resolution observed in the conditioned-media measurements may reflect compartment-specific signaling (intracellular and EV-encoded reprogramming preceding measurable secreted cytokine changes), differences in temporal kinetics, or the limited dynamic range of the cytokine panel. We have flagged this discordance explicitly throughout the manuscript and emphasize that the proteomic findings should be interpreted as molecular states encoded in the EV cargo rather than as direct evidence of resolved tissue inflammation.

## 5. Conclusions

This study presents the first comprehensive characterization of CSE effects on CTC-derived EV cargo and the first systematic comparison of prophylactic, therapeutic, and combined aspirin regimens across apoptotic, angiogenic, and inflammatory pathway domains. These EV markers may offer a tangible path toward precision obstetrics, moving beyond the “one-size-fits-all” approach to identify aspirin-responsive pregnancies via non-invasive liquid biopsy. Ultimately, incorporating the chorion into our pharmacological models redefines our understanding of preeclampsia prophylaxis, shifting the focus from simple placental perfusion to holistic feto-maternal barrier defense. Future translational studies integrating PTC and CTC-EV networks, EV profiling, microphysiological models, and maternal plasma biomarker validation will further determine how fetal membrane dynamics affect the overall therapeutic landscape of aspirin in pregnancy.

## Figures and Tables

**Table 1:** Prophylactic EV characterization by Zeta View.

	Concentration (Particles/mL)		Size (nm)	
	Mean	SD	Median	SD
**CTC Control**	2.30 × 10^10^	2.0 × 10^9^	119.5	44.1
**CTC_CSE**	4.5 × 10^10^	6.0 × 10^9^	105.2	40.3
**CTC_Asp4_CSE**	5.8 × 10^10^	6.7 × 10^9^	107.8	46.3
**CTC_Asp40_CSE**	3.4 × 10^10^	4.0 × 10^9^	110.3	45.5
**CTC_Asp4_CSE+Asp4**	4.8 × 10^10^	1.2 × 10^10^	105.7	42
**CTC_Asp40_CSE+Asp40**	4.9 × 10^10^	5.5 × 10^9^	110.0	42.5

**Table 2:** Therapeutic EV characterization by Zeta View.

	Concentration (Particles/mL)		Size (nm)	
	Mean	SD	Median	SD
**CTC Control**	2.60 × 10^10^	1.90 × 10^9^	141.7	61.9
**CTC_Asp4**	1.40 × 10^10^	1.2 × 10^9^	137.0	61.4
**CTC_Asp40**	2.60 × 10^10^	2.20 × 10^9^	135.8	58.0
**CTC_CSE**	2.40 × 10^10^	2.10 × 10^9^	142.4	64.4
**CTC_CSE+Asp4**	3.50 × 10^10^	3.60 × 10^9^	139.8	66.7

**Table 3:** CSE-induced damage signature proteins.

Protein	Direction	Functional Significance
F10 (Factor X)	↑↑↑ in CSE	Pro-coagulant EV cargo; thrombotic risk
CYP1A1	↑ in CSE	Xenobiotic metabolism; smoking biomarker
MFG-E8	↓↓ in CSE	Lost efferocytosis and anti-inflammatory signaling
VPS4B	↓ in CSE	Disrupted ESCRT/EV biogenesis
EFNB2	↓ in CSE	Lost vascular guidance signaling

↑, upregulated; ↓, downregulated; ↑↑↑, strongly upregulated; ↓↓↓, strongly downregulated relative to control.

**Table 4:** Functional outcome of prophylactic low or high dose of aspirin.

Functional Outcome	Asp4-CSE (Low Dose)	Asp40-CSE (High Dose)
Reduced apoptosis vs. CSE	Yes	Yes
Restored angiogenesis vs. CSE	Yes	Not observed
Inflammatory pathway modulation	Yes	Partial
Pro-angiogenic protein enrichment	LGR4, THY1, CAV1, MMP14, MYOF	Absent

**Table 5:** Functional outcome of continuous prophylactic low or high dose of Aspirin.

Functional Outcome	Asp4-CSE-Asp4 (Low Continuous)	Asp40-CSE-Asp40 (High Continuous)
Reduced apoptosis vs. CSE	Yes	Yes
Initiation of angiogenesis	Yes (strong)	Yes (partial)
Antioxidant pathway enrichment	GSTM3 upregulated	Less prominent
EV biogenesis markers	ATPV0D1 upregulated	Less clear
Vesicular trafficking	RABL3, RAP2C	MARCKS, MARCKSL1

**Table 6:** Comparative analysis of prophylactic and therapeutic treatments.

Treatment Paradigm	Apoptosis Rescue	Angiogenesis Rescue	Overall Assessment
CSE alone	Increased apoptosis	Reduced angiogenesis	Damage baseline
Asp4-CSE (Prophylactic low)	Reduced	Increased	Best co-treatment
Asp40-CSE (Prophylactic high)	Reduced	Not observed	Partial protection
Asp4-CSE-Asp4 (Continuous low)	Reduced	Initiated	Best overall
Asp40-CSE-Asp40 (Continuous high)	Reduced	Initiated (partial)	Good protection

**Table 7:** Pro-angiogenic protein regulators observed in this study.

Protein	Function	Condition Observed
LGR4	Wnt signaling receptor; promotes endothelial proliferation	Asp4-CSE ↑
THY1 (CD90)	Cell surface glycoprotein; angiogenesis and adhesion	Asp4-CSE ↑
CAV1 (Caveolin-1)	Membrane scaffolding; VEGF signaling	Asp4-CSE ↑
MMP14 (MT1-MMP)	Extracellular matrix remodeling; invasion	Asp4-CSE ↑
MYOF (Myoferlin)	Membrane repair; VEGFR2 signaling; angiogenesis	Asp4-CSE ↑
RHOA	Rho GTPase; endothelial migration	Asp4-CSE ↑, Asp40-CSE ↑
SLC4A7	Bicarbonate transporter; pH regulation in angiogenesis	Asp4-CSE ↑
MARCKS	Membrane dynamics; cell motility	Asp4-CSE-Asp4 ↑, Asp40-CSE-Asp40 ↑
GSTM3	Antioxidant defense; prevents oxidative damage	Asp4-CSE-Asp4 ↑
ATPV0D1	Vacuolar ATPase; endosomal function; EV biogenesis	Asp4-CSE-Asp4 ↑

↑, upregulated; ↓, downregulated; ↑↑↑, strongly upregulated; ↓↓↓, strongly downregulated relative to control.

## Data Availability

The original contributions presented in this study are included in the article/Supplementary Materials. Further inquiries can be directed to the corresponding author.

## Supplementary Materials

The following supporting information can be downloaded at: https://www.mdpi.com/article/10.3390/pharmaceutics18060677/s1.
