# Supplementary material for: From Stress to Survival: Trophoblast-Derived Extracellular Vesicle Proteome Captures Aspirin-Driven Cellular Reprogramming in a Preeclampsia Model
Source: Pharmaceutics. 2026 May 29;18(6):677. doi: 10.3390/pharmaceutics18060677 (PMC13305446; doi:10.3390/pharmaceutics18060677)
Supplement: Supplementary file 1 [file pharmaceutics-18-00677-s001.zip › pharmaceutics-4267205-supplementary.pdf]

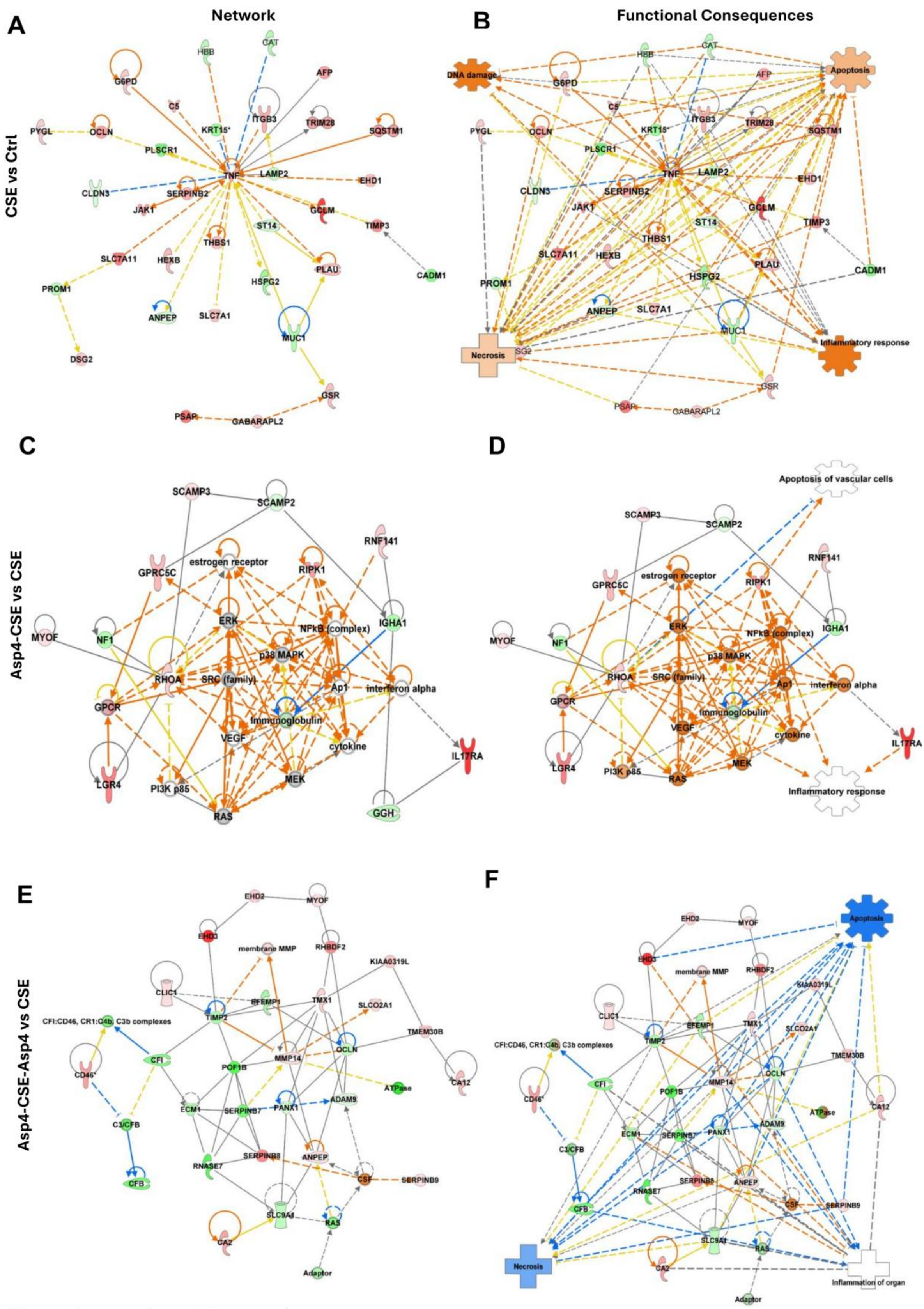

Supplementary Figure 1

Figure S1: IPA identifies distinct signaling networks driving prophylactic Aspirin-induced Cell Survival.

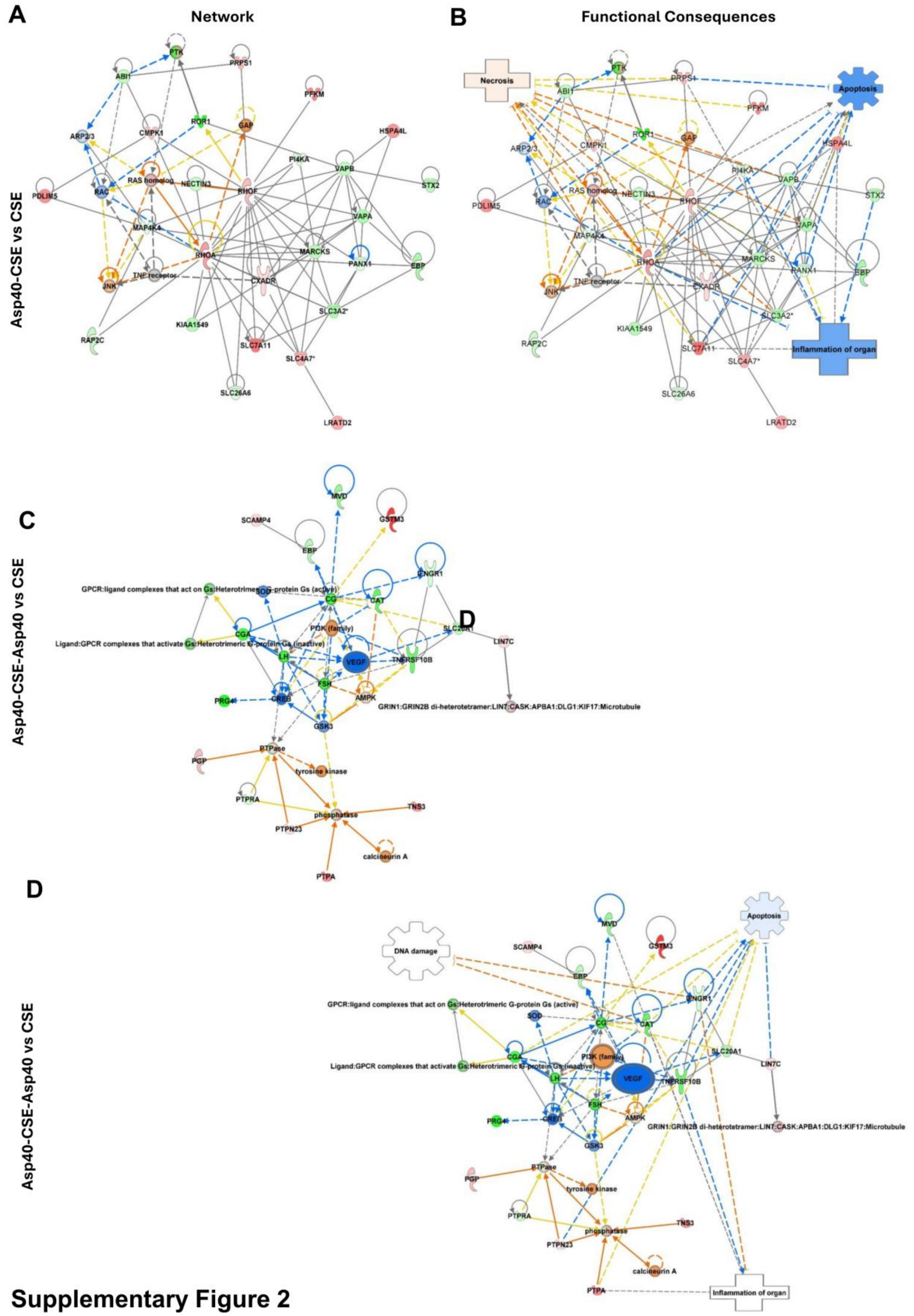

Supplementary Figure 2

Figure S2: Sequential aspirin treatment (pre- and post-CSE) reprograms DNA damage, cell death, and inflammatory signaling.

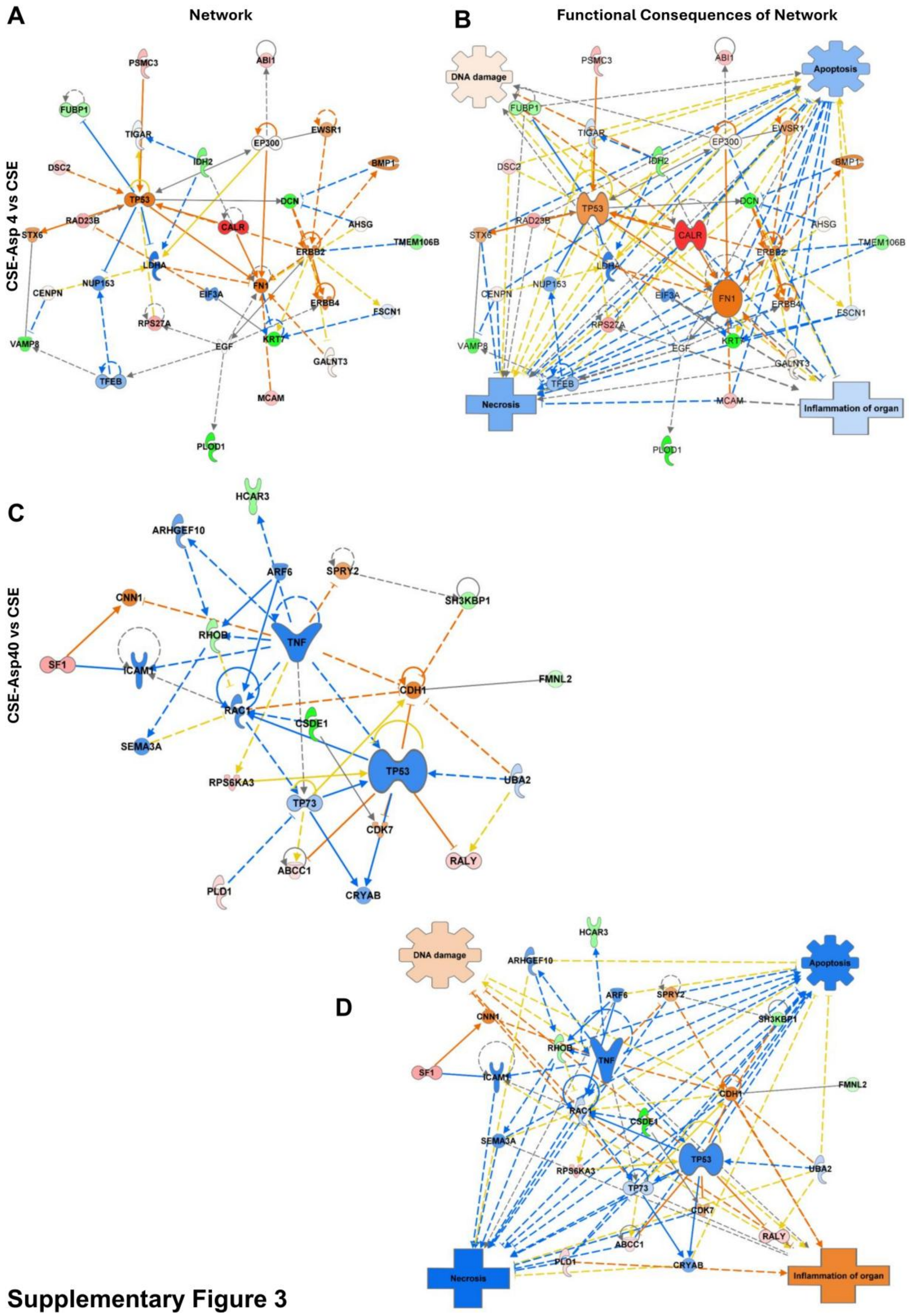

Supplementary Figure 3

Figure S3: Therapeutic aspirin mitigates CSE-induced cellular injury via network-level reprogramming.

Therapeutic	Concentration (Particles/ml)		Size (nm)	
	Mean	SD	Median	SD
CTC Control	2.60E+10	1.90E+09	141.7	61.9
CTC_Asp4	1.40E+10	1.2E+9	137.0	61.4
CTC_Asp40	2.60E+10	2.20E+09	135.8	58.0
CTC_CSE	2.40E+10	2.10E+09	142.4	64.4
CTC_CSE+Asp4	3.50E+10	3.60E+09	139.8	66.7
CTC_CSE+Asp40	2.10E+10	1.50E+09	138.0	54.1

Sample Name: CTC_Asp4_2_1

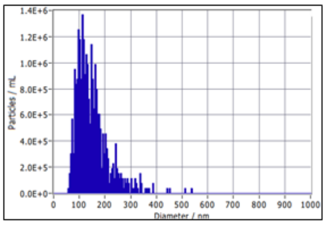

Result (sizes in nm)

	Number	Concentration	Volume
Median (X50)	132.2	132.2	240.9
StdDev	65.7	65.7	115.0

Concentration: 2.6E+7 Particles / mL
Dilution Factor: 500
Concentration Correction Factor: 1.00000
Original Concentration: 1.3E+10 Particles / mL

Quality
Average Counted Particles per Frame: 61
Number of Traced Particles: 673

Analysis Parameters
Max Area 1000, Min Area 10, Min Brightness 20, nm/Class: 5

Prophylactic	Concentration (Particles/ml)		Size (nm)	
	Mean	SD	Median	SD
CTC Control	2.30E+10	2.0E+9	119.5	44.1
CTC_CSE	4.5E+10	6.0E+9	105.2	40.3
CTC_Asp4_CSE	5.8E+10	6.7E+9	107.8	46.3
CTC_Asp40_CSE	3.4E+10	4.0E+9	110.3	45.5
CTC_Asp4_CSE+Asp4	4.8E+10	1.2E+10	105.7	42
CTC_Asp40_CSE+Asp40	4.9E+10	5.5E+9	110.0	42.5

Sample Name: CTC_Asp40_2_2

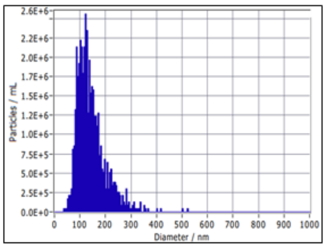

Result (sizes in nm)

	Number	Concentration	Volume
Median (X50)	127.1	127.1	214.2
StdDev	57.5	57.5	100.7

Concentration: 4.6E+7 Particles / mL
Dilution Factor: 500
Concentration Correction Factor: 1.00000
Original Concentration: 2.3E+10 Particles / mL

Quality
Average Counted Particles per Frame: 109
Number of Traced Particles: 1069

Analysis Parameters
Max Area 1000, Min Area 10, Min Brightness 20, nm/Class: 5

Supplementary Figure 4
