# Supplementary figures and images for: From Stress to Survival: Trophoblast-Derived Extracellular Vesicle Proteome Captures Aspirin-Driven Cellular Reprogramming in a Preeclampsia Model
Source: Pharmaceutics. 2026 May 29;18(6):677. doi: 10.3390/pharmaceutics18060677 (PMC13305446; doi:10.3390/pharmaceutics18060677)

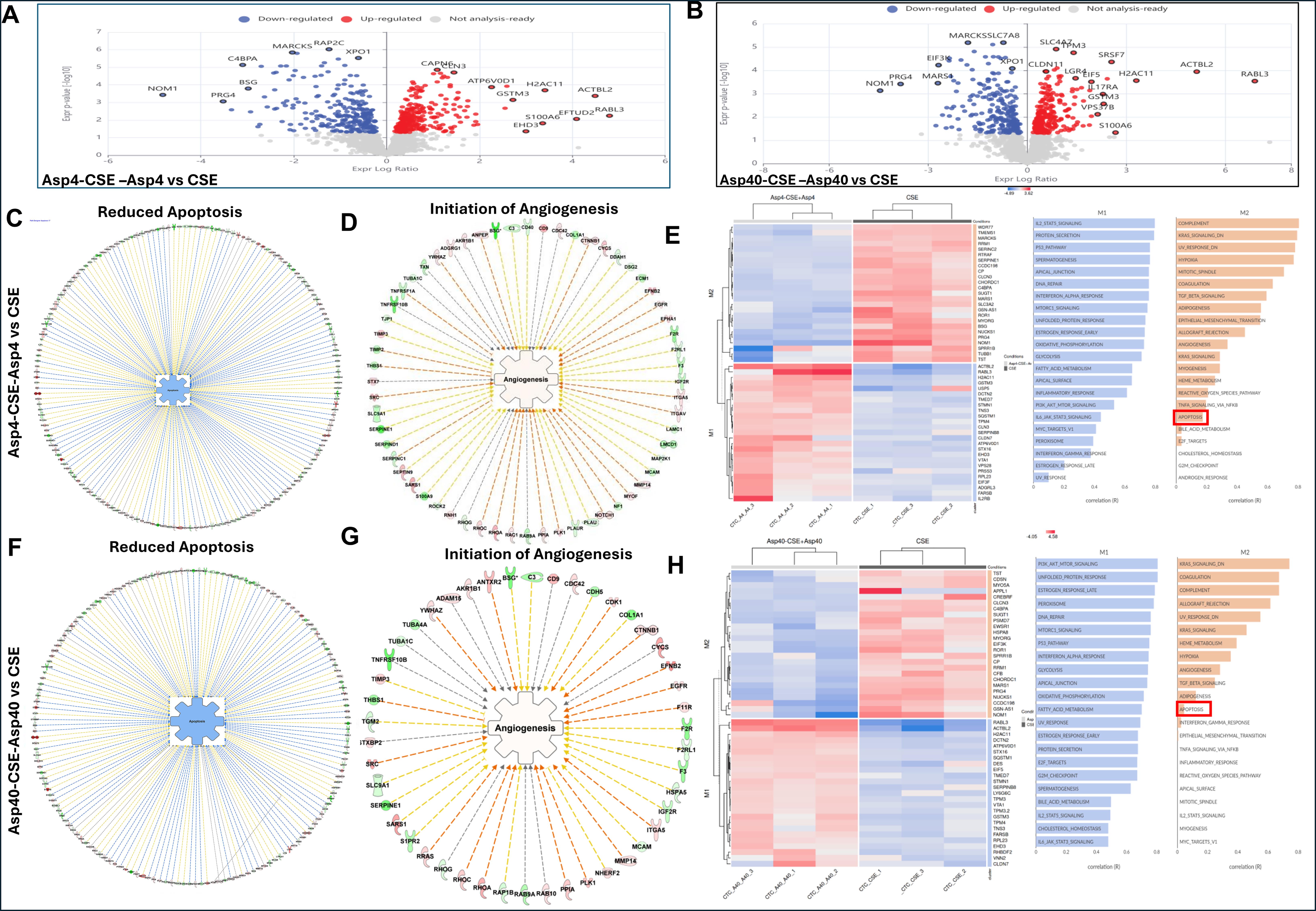

Supplement: Supplementary file 1 [file pharmaceutics-18-00677-s001.zip › High-resolutionof Figure 5.jpg]
